# Bioinspired Multifunctional Self-Sensing Actuated Gradient Hydrogel for Soft-Hard Robot Remote Interaction

**DOI:** 10.1007/s40820-023-01287-z

**Published:** 2024-01-04

**Authors:** He Liu, Haoxiang Chu, Hailiang Yuan, Deliang Li, Weisi Deng, Zhiwei Fu, Ruonan Liu, Yiying Liu, Yixuan Han, Yanpeng Wang, Yue Zhao, Xiaoyu Cui, Ye Tian

**Affiliations:** 1 College of Medicine and Biological Information Engineering, Northeastern University, Shenyang, 110169 People’s Republic of China; 2 Foshan Graduate School of Innovation, Northeastern University, Foshan, 528300 People’s Republic of China

**Keywords:** Self-sensing, Gradient structure, Bioinspired actuator, Hydrogel sensor, Remote interaction

## Abstract

The development of bioinspired gradient hydrogels with self-sensing actuated capabilities for remote interaction with soft-hard robots remains a challenging endeavor. Here, we propose a novel multifunctional self-sensing actuated gradient hydrogel that combines ultrafast actuation and high sensitivity for remote interaction with robotic hand. The gradient network structure, achieved through a wettability difference method involving the rapid precipitation of MoO_2_ nanosheets, introduces hydrophilic disparities between two sides within hydrogel. This distinctive approach bestows the hydrogel with ultrafast thermo-responsive actuation (21° s^−1^) and enhanced photothermal efficiency (increase by 3.7 °C s^−1^ under 808 nm near-infrared). Moreover, the local cross-linking of sodium alginate with Ca^2+^ endows the hydrogel with programmable deformability and information display capabilities. Additionally, the hydrogel exhibits high sensitivity (gauge factor 3.94 within a wide strain range of 600%), fast response times (140 ms) and good cycling stability. Leveraging these exceptional properties, we incorporate the hydrogel into various soft actuators, including soft gripper, artificial iris, and bioinspired jellyfish, as well as wearable electronics capable of precise human motion and physiological signal detection. Furthermore, through the synergistic combination of remarkable actuation and sensitivity, we realize a self-sensing touch bioinspired tongue. Notably, by employing quantitative analysis of actuation-sensing, we realize remote interaction between soft-hard robot via the Internet of Things. The multifunctional self-sensing actuated gradient hydrogel presented in this study provides a new insight for advanced somatosensory materials, self-feedback intelligent soft robots and human–machine interactions.

**Supplementary Information:**

The online version contains supplementary material available at 10.1007/s40820-023-01287-z.

## Highlights

- The bioinspired self-sensing actuated gradient hydrogel was developed by a wettability-based method via precipitation of MoO_2_ nanosheets.
- Self-sensing actuated gradient hydrogel combined ultrafast thermo-responsive actuation (21° s^–1^), exceptional photothermal efficiency (3.7 °C s^–1^) and high sensing properties (GF = 3.94).
- The first self-sensing remote interaction system based on gradient hydrogel actuators and robotic hands was constructed.

**Supplementary Information:**

The online version contains supplementary material available at 10.1007/s40820-023-01287-z.

## Graphical abstract

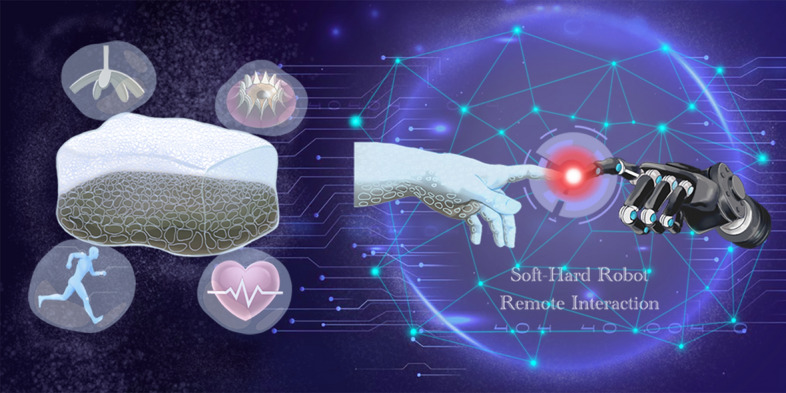

## Introduction

Nature provides a great deal of inspiration for the development of soft robots [1, 2]. Many living organisms, such as human tongue, jellyfish tentacles, and mimosa leaves, can not only deform flexibly over long distances, but also possess self-sensing capabilities. To mimic these natural systems, researchers have developed bioinspired soft robots by combining soft actuators and flexible sensors [3, 4]. However, a prevailing limitation is that most soft actuators, while capable of complex shapes, lack the ability to sense external stimuli and monitor their own movements. As a remedy, researchers have resorted to embedding sensors, leading to increased complexity in soft robot design and manufacturing. Consequently, the application of soft robots in environmental interactions is hindered. In light of these challenges, the development of bioinspired multifunctional self-sensing soft robots has emerged as a compelling and relevant research direction.

Stimulus-responsive hydrogels, representing a novel class of intelligent soft materials, have garnered considerable attention in various fields, including soft robotics [5, 6], sensors [7, 8], artificial muscles [9, 10] and biomedical engineering [11–13]. These hydrogels can be flexibly deformed by responding to external environmental stimuli such as temperature [14, 15], near-infrared (NIR) light [16, 17], pH [18, 19], humidity [20, 21], and electric [22] or magnetic fields [23]. Among these stimuli, NIR-responsive hydrogel actuators have emerged as particularly promising due to their superior spatial and temporal resolution. Recently, researchers have introduced photothermally convertible conductive polymers [24, 25] or conductive nanoparticles [26–28] into hydrogels, resulting in the development of self-sensing hydrogel actuators capable of both NIR-responsive actuation and conductive sensing. For instance, conductive hydrogels were prepared by incorporating the conductive polymer polyaniline (PANI) into a dual network poly(N isopropylacrylamide-co-acrylamide)/poly(vinyl alcohol) (PNA/PVA) hydrogel [25]. By combining with passive polyacrylamide (PAAM) layers, bilayer self-sensing hydrogel actuators were fabricated, where changes in electrical resistance allow real-time sensing of the actuation state. However, these bilayer hydrogel actuators exhibited relatively poor actuation speed and sensing properties and were prone to delamination over prolonged use. A homogeneous somatosensory hydrogel actuator was prepared by in situ copolymerization of conductive surface-functionalized MXene (K-MXene)/ PEDOT:PSS ink with thermos-responsive PNIPAM hydrogels, achieving fast, shape programmable NIR-responsive actuation and good self-sensing [26]. Nevertheless, homogeneous hydrogel actuators posed challenges in maintaining precise control of actuation behavior over extended periods due to the need for a continuous gradient of stimuli. Gradient hydrogel actuators have been extensively investigated due to their integrated, flexibly controlled and smooth continuous changes under external stimuli [29–32]. However, self-sensing hydrogel actuators based on gradient structures have not yet been developed. Notably, the above existing self-sensing hydrogel actuators were mainly concerned with their sensing behavior, lack quantification of sensing information, and do not yet enable soft-hard robotic interaction. Therefore, it is urgently demanding to fabricate self-sensing gradient hydrogel actuator for robotic remote interaction.

**Fig. 1:**
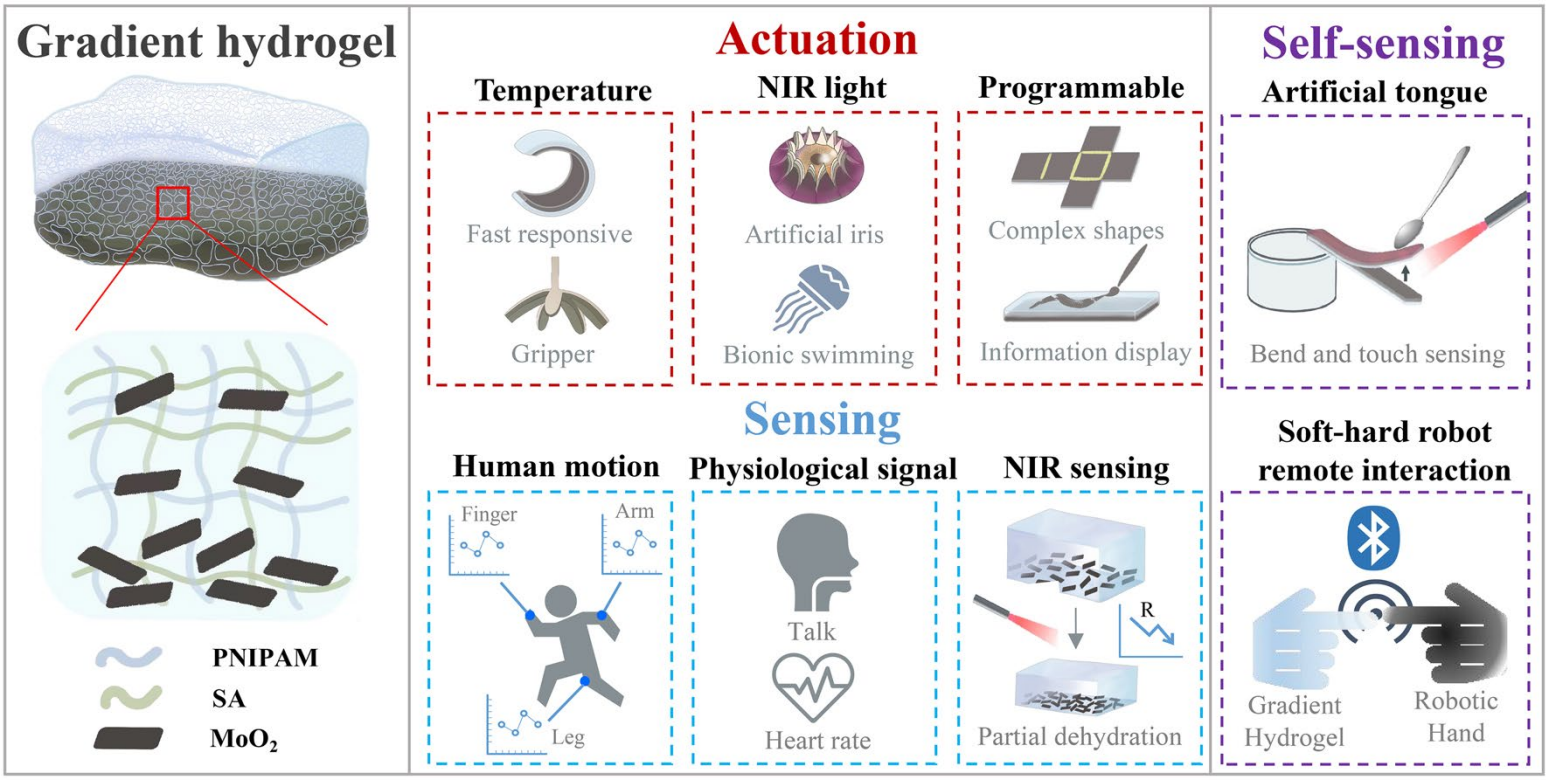
Schematic illustration of the synthesis, properties and applications of self-sensing actuated gradient hydrogel

Herein, we presented a facile wettability-based method to prepare a bioinspired multifunctional self-sensing actuated gradient hydrogel with both ultrafast actuation and high sensing for soft-hard robot remote interaction (Fig. 1). The difference in wettability caused by introducing MoO_2_ nanosheets in the copolymerization of the N-isopropylacrylamide (NIPAM) and sodium alginate (SA) monomer, resulting in gradient hydrogel. This innovative approach allows the resulting hydrogel to achieve ultrafast actuation, with bending speed reached 21° s^−1^ in 50 °C water, which can be adjusted by controlling the soaking time of Ca^2+^, the MoO_2_ content and the thickness of the hydrogel. As a practical application, we have developed a soft gripper, reminiscent of the game Gold Miner, which can rapidly grip target objects within 20 s. Moreover, the hydrogels have an exceptional photothermal efficiency (3.7 °C s^−1^) under NIR irradiation (808 nm, 2 W cm^−2^). Leveraging this property, we designed light-responsive bioinspired soft robots including artificial irises and jellyfish. Meanwhile, by locally cross-linking Ca^2+^, we achieved multiple programmable deformations. Information display and hiding was achieved by combining the volume phase transition of PNIPAM-based hydrogels under thermal stimulation, and information can be erased by Ethylenediaminetetraacetic acid (EDTA) solutions. In addition, the hydrogels demonstrated remarkable sensing properties, featuring high sensitivity (GF = 3.94) within a wide strain range (600%), fast response times (140 ms) and recycling stability. As a result, we successfully implemented precise human motion detection (finger, wrist, arm and leg), and physiological signal detection (speech and ECG). Combining the unique actuation and sensing properties of hydrogels, we have achieved a bioinspired tongue that is self-sensing to bend and touch. Significantly, by integrating Internet of Things (IoT) technology, we achieved remote interactions between soft and hard robots. We quantified the relationship between real-time resistance and bending angle of the self-sensing hydrogel actuator. The first self-sensing remote interaction system based on gradient hydrogel actuators and robotic hands was constructed. Our bioinspired multifunctional self-sensing actuated gradient hydrogel opens up new avenues in the design of advanced somatosensory materials, with promising applications in soft robotics, wearable electronics and human–machine interaction.

## Experimental Section

### Materials

*N*-Isopropylacrylamide (NIPAM), Molybdenum oxide (MoO_2_), Sodium alginate (SA), *N*,*N′*-methylene bisacrylamide (BIS), potassium persulfate (KPS), and *N*,*N*,*N′*,*N′*-tetramethylethylenediamine (TEMED) were all purchased from Aladdin Chemical Reagent Co. Deionized (DI) water from a water purification system (UPTA-UV-20, Shenfen, China) was used throughout the experiments. The reagents were used as received unless otherwise noted.

### Preparation of PSM Hydrogels

PSM hydrogels were prepared by the in situ copolymerization of NIPAM and SA monomers in a dispersion of MoO_2_. Typically, NIPAM (1.6 g) and BIS (2 mg) were first dissolved in DI water (12 mL) under stirring for 10 min. Then SA (0.12 g) was added into the solution under stirring for 1 h. Afterward, MoO_2_, KPS (40 mg), and TEMED (9 μL) were added to the solution under stirring for 10 min at 10 °C. Finally, the solution was quickly placed into 50 mm × 10 mm × *T* mm silicone molds, where *T* stood for the thickness of mold (*T* = 1, 2, or 3), and polymerized at 10 °C for 4 h. Here, the prepared hydrogels were named PSM_x_, where x stood for the content of MoO_2_ (*x* = 0.2, 0.4, 0.6 or 0.8 g). As a control, PSM hydrogels without MoO_2_ are named PS and pure PNIPAM hydrogels are called P.

### Characterizations of PSM Hydrogels

Microstructures of resultant hydrogels were observed by scanning electron microscopy (SEM, SEM5000, CIQTEK, China) at an accelerating voltage of 5 kV. Before the measurement, hydrogels were frozen in liquid nitrogen and then dried in freeze dryer (FD-A12N-80, Guansen Biotechnology, China) for 24 h. Fourier transform infrared (FTIR) spectroscopy measurements were performed on a spectrometer (VERTEX70, Bruker, Germany) in the scanning range from 500 to 4000 cm^−1^. The mechanical tests were measured using Universal Electronic Tester (Model 43, MTS Criterion, US), where hydrogel samples (10 mm × 10 mm × 1 mm) were used in the tensile tests with a velocity of 50 mm min^−1^. The volume phase transition temperature (VPTT) of hydrogel was measured on a differential scanning calorimeter (DSC, 404F3, Bruker, Germany) by fast cooling samples to −10 °C, followed by reheating to 50 °C at a scanning rate of 5 °C min^−1^. The real-time resistances of hydrogels were tested using electrochemical workstation (760E, CHI, US) with the alternating current (AC) voltage 1 V and the AC frequency 1000 Hz.

## Result and Discussion

### Fabrication and Characterization of PSM Hydrogel

**Fig. 2:**
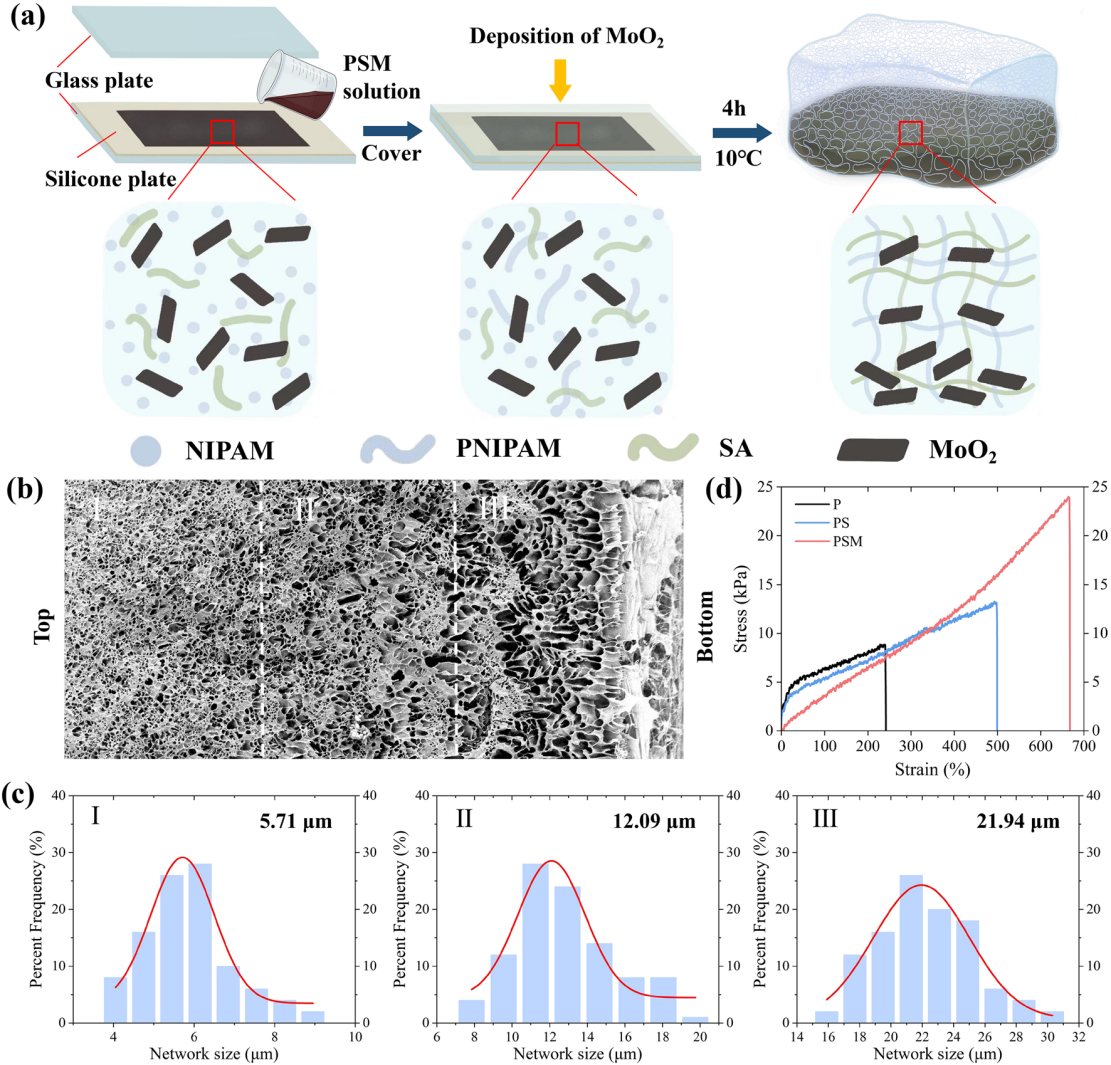
Synthesis and characterization of PSM hydrogels. **a** Schematic illustration of the synthesis, polymerization dispersion, and gradient network structure of PSM hydrogel. **b** SEM images of PSM hydrogel with large-ranged gradient structure. **c** Network size statistics of PSM hydrogel at three different locations in **b**. **d** Stress–strain curves of P, PS and PSM hydrogel

PSM Gradient hydrogels were fabricated by in situ copolymerization of SA, MoO_2_ and thermosensitive PNIPAM hydrogels. The specific preparation process was shown in Fig. 2a. Initially, a mixed solution of SA, MoO_2_ and NIPAM monomer was poured into the mold, and then the glass plate was covered horizontally. During the polymerization process, the MoO_2_ flakes rapidly settled to the bottom of the mixed solution under the effect of gravity. Given the hydrophobicity of MoO_2_ flakes, the hydrophilic NIPAM monomer is more easily attracted to the top, leading to an abundant PNIPAM phase polymerization. Consequently, the hydrogel acquired a gradient structure along the direction of gravity. SEM images confirmed the distinct gradient network structure of the PSM hydrogels (Fig. 2b), with the network size increased from 5.71 μm in region I to 21.94 μm in region III (Fig. 2c). This gradient structure induces different degrees of shrinkage and deformation of the PSM hydrogel in response to external stimuli. FTIR analysis of the characteristic peaks verified the chemical bonding (Fig. S1). The broad characteristic peak at 3470 cm^−1^ of PSM was attributed to the superposition of the -OH absorption peak (3370 cm^−1^) of SA and the N–H absorption peak (3280 cm^−1^) of NIPAM. The peak at 1640 cm^−1^ of PSM was considered to the C = O stretching peak in amide I (1660 cm^−1^) of NIPAM. The introduction of MoO_2_ flakes did not affect the formation of characteristic peaks. These results suggest that PSM hydrogels have an interpenetrating network of alginate and PNIPAM. In addition, the mechanical properties of the PSM hydrogels were investigated (Fig. 2d). The results showed that the tensile strain and stress properties of the hydrogels were improved by the introduction of SA and MoO_2_, where the PSM_0.6_ hydrogel had the best fracture strain of 666% and the best fracture stress of 23.9 kPa. Such low Young's modulus (3.9 kPa) and high tensile strain property enables ultrafast actuation of the PSM hydrogels.

### Thermo-responsive Actuation of PSM Hydrogel

**Fig. 3:**
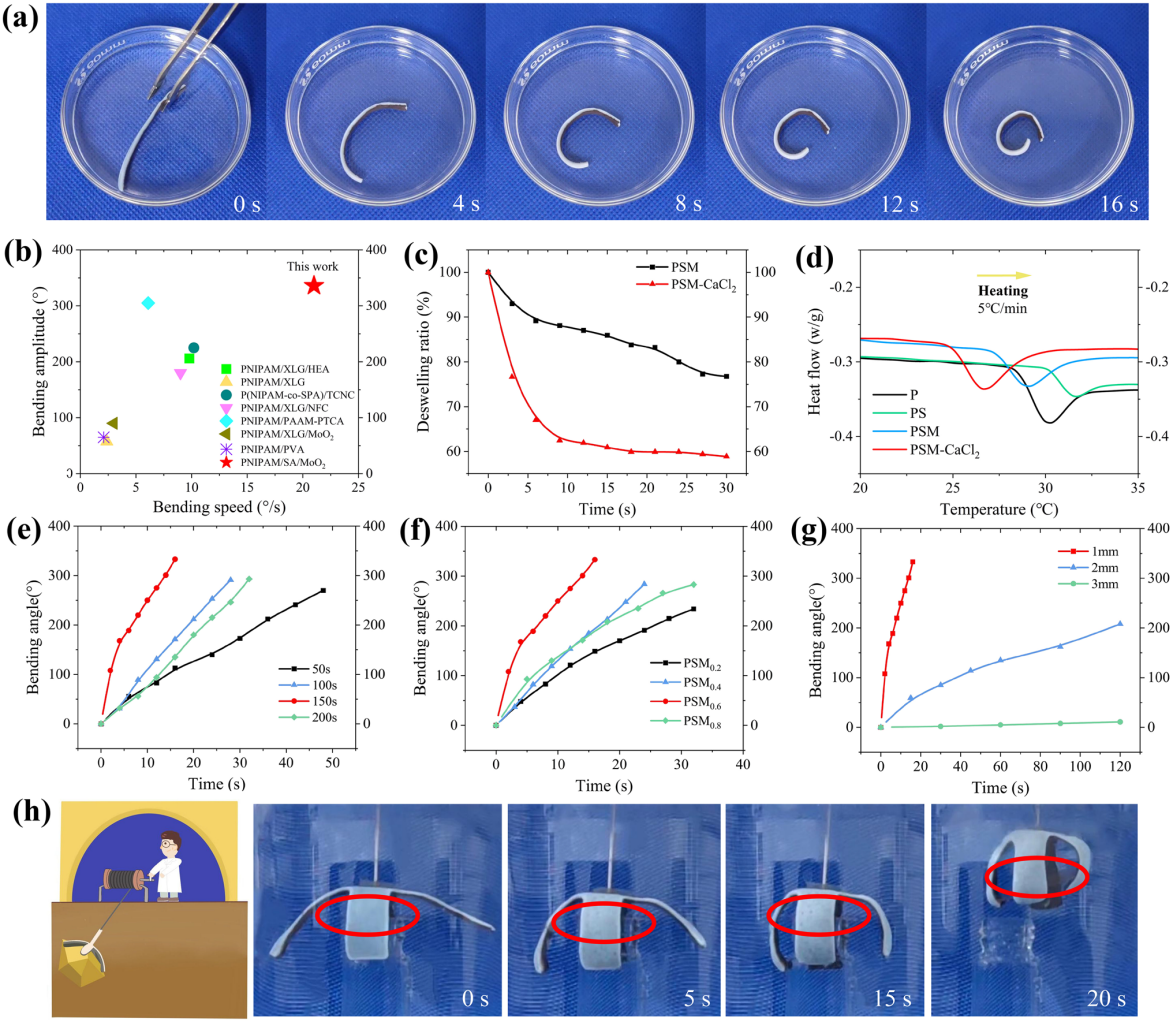
Thermo-responsive actuation of PSM hydrogels. **a** Bending behavior of PSM hydrogel with a thickness of 1 mm in water at 50 °C. **b** Comparison of bending speed and bending amplitude for different hydrogel actuators under external stimuli. The detailed information is shown in Table S1. **c** Deswelling curve of PSM and PSM-CaCl_2_ hydrogel in water at 50 °C. **d** DSC curves of P, PS, PSM and PSM-CaCl_2_ hydrogel. **e** Effect of CaCl_2_ soaking time on bending response in water at 50 °C. **f** Effect of MoO_2_ content in hydrogels on bending response in water at 50 °C. **g** Effect of the thickness of the hydrogel on bending response in water at 50 °C. **h** Process of PSM hydrogel gripper grabbing metal sheet from 50 °C water

Taking advantage of the asymmetric gradient structure of PSM hydrogels, ultrafast thermo-responsive hydrogel actuators were prepared by immersing PSM in CaCl_2_. The actuation properties of the hydrogel were evaluated by measuring the bending angle of the hydrogel in water at 50 °C (as defined in Fig. S2). Remarkably, the CaCl_2_-soaked PSM hydrogel can bend into a circle (336°) toward the top side at 21° s^−1^ (Fig. 3a and Movie S1). This actuation performance exceeds most reported thermos-responsive hydrogel actuators (Fig. 3b and Table S1). The enhanced actuation speed can be attributed to the larger network size at the bottom of the MoO_2_-rich hydrogel, which allows for greater shrinkage and results in a rapid thermal response. The actuation speed was 3.4 times faster than the PSM hydrogel without CaCl_2_ soaking (Fig. S3). The reason is probably that the CaCl_2_ soaking caused significant increase in the deswelling rate of the hydrogel (Fig. 3c), indicating more rapid and extensive volume reduction at 50 °C. Also, the introduction of CaCl_2_ changed the volume phase transition temperature (VPTT) of hydrogels. The DSC measurements showed that PSM hydrogels had a lower VPTT compared to pure PNIPAM hydrogels, and the addition of CaCl_2_ further reduced the VPTT of the hydrogel (Fig. 3d), leading to an even faster response rate.

We further investigated the key factors affecting the performance of hydrogel actuators, including CaCl_2_ soaking time, MoO_2_ content and hydrogel thickness. The deswelling rate of PSM hydrogels was influenced by the CaCl_2_ soaking time, with 150 s being the fastest rate (Fig. S4). As shown in Figs. 3e and S5, the results demonstrated the hydrogel with CaCl_2_ soaked for 150 s exhibited the fastest bending speed. The bending speed of the PSM with CaCl_2_ soaked for 200 s was reduced, potentially due to excessive cross-linking of CaCl_2_ over a long time, leading to a large increase in the Young's modulus of the hydrogel (Fig. S6). Moreover, MoO_2_ content in the hydrogel significantly affected the bending speed, where the PSM_0.6_ hydrogel has the fastest bending speed (Fig. 3f). Increasing MoO_2_ content up to 0.6 g enhanced the bending speed (Figs. S7a, b and 3a). However, an excessive MoO_2_ content (0.8 g) obstructed the network at the bottom of the hydrogel, affecting water discharge and resulting in a decrease in bending speed (Fig. S7c). This observation was supported by the notable decrease in the deswelling rate of PSM_0.8_ (Fig. S8). Furthermore, the thickness of the hydrogel has a significant effect on the actuation behavior. As shown in Figs. 3g and S9, the bending speed decreases with increasing hydrogel thickness in 50 °C water, mainly because thicker hydrogels require larger forces for actuation. Taking advantage of the ultrafast thermo-response actuation properties of PSM hydrogels, we realized the fabrication of a bioinspired four-armed gripper (like Gold Miner) to achieve grasping a target object (Fig. 3h and Movie S2). When placed in 50 °C water, the gripper rapidly bent downward and grasped the metal sheet within 15 s.

### Light-responsive Actuation of PSM Hydrogel

**Fig. 4:**
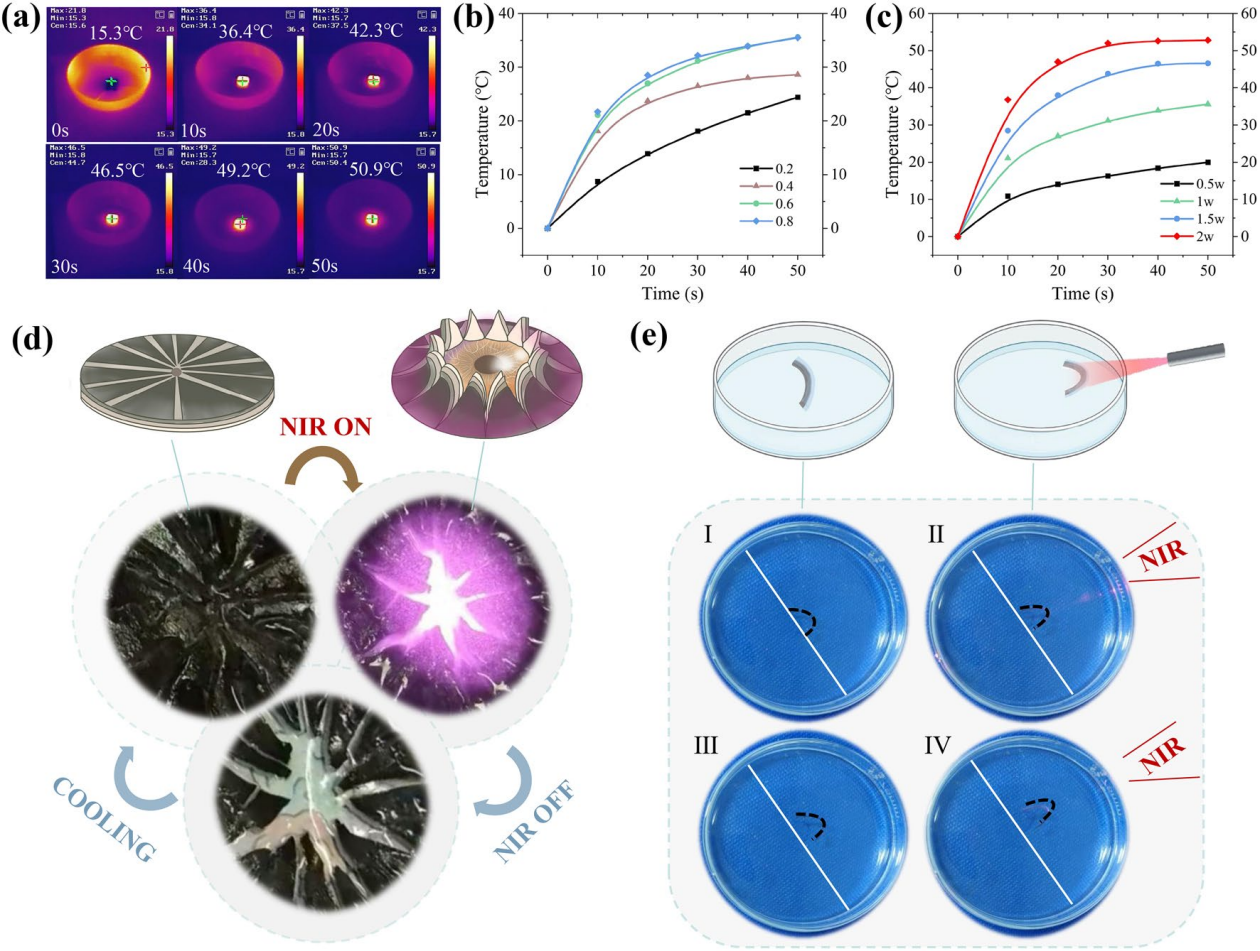
Light-responsive actuation of PSM hydrogels. **a** Temperature changes of PSM_0.6_ hydrogel under 808 nm NIR light irradiation with 1 W cm^−2^. **b** Temperature response curves of the PSM hydrogels with various MoO_2_ contents under NIR light irradiation (808 nm, 1 W cm^−2^). **c** Temperature response curves of the PSM_0.6_ hydrogel with different power densities under 808 nm laser. **d** Schematic illustration and photos of an artificial iris. **e** Schematic illustration of bioinspired jellyfish swimming under a NIR switch

Due to the NIR photothermal effect of MoO_2_, the temperature of PSM hydrogel will increase with the irradiation of NIR light. As shown in Fig. 4a, the temperature of the PSM hydrogel can reach 36.4 °C after the NIR light irradiation (808 nm, 1 W cm^−2^) for 10 s. The MoO_2_ content has an impact on the photothermal effect, and Figs. 4b and S10 show PSM hydrogels with higher MoO_2_ content resulting in improved photothermal conversion. In particular, PSM_0.6_ and PSM_0.8_ have similar temperature rise rates, reaching nearly 2.2 °C s^−1^ within 10 s of NIR light irradiation (808 nm, 1 W cm^−2^). Moreover, the temperature rise rate of the PSM hydrogel increases with higher laser power density (Figs. 4c and S11), reaching nearly 3.7 °C s^−1^ within 10 s of NIR light irradiation (808 nm, 2 W cm^−2^). Thus, PSM gradient hydrogels demonstrated good light-responsive actuation due to the hydrogel can achieve its LCST in a short time under NIR light irradiation.

Taking advantage of these light-responsive properties, we successfully fabricated a bioinspired artificial iris using PSM hydrogels. As shown in Fig. 4d, the iris gradually opened under NIR light irradiation, revealing a background pattern of flowers, and could be closed after turning off the NIR light. Furthermore, the actuation behavior showed good recyclability. In addition, a light-responsive soft robot inspired by the swimming movement of jellyfish was developed (Fig. 4e and Movie S3). The PSM gradient hydrogel was placed on the surface of the water and when the NIR was irradiated, the gradient hydrogel swam due to fluid flow generated by thermal shrinkage and deformation. And when the NIR light was turned off, the hydrogel dissolved in the water to return to its original state. By cyclically turning the NIR light on and off, PSM hydrogel swam in the direction of the light.

### Programmable Deformation and Information Display of PSM Hydrogel

**Fig. 5:**
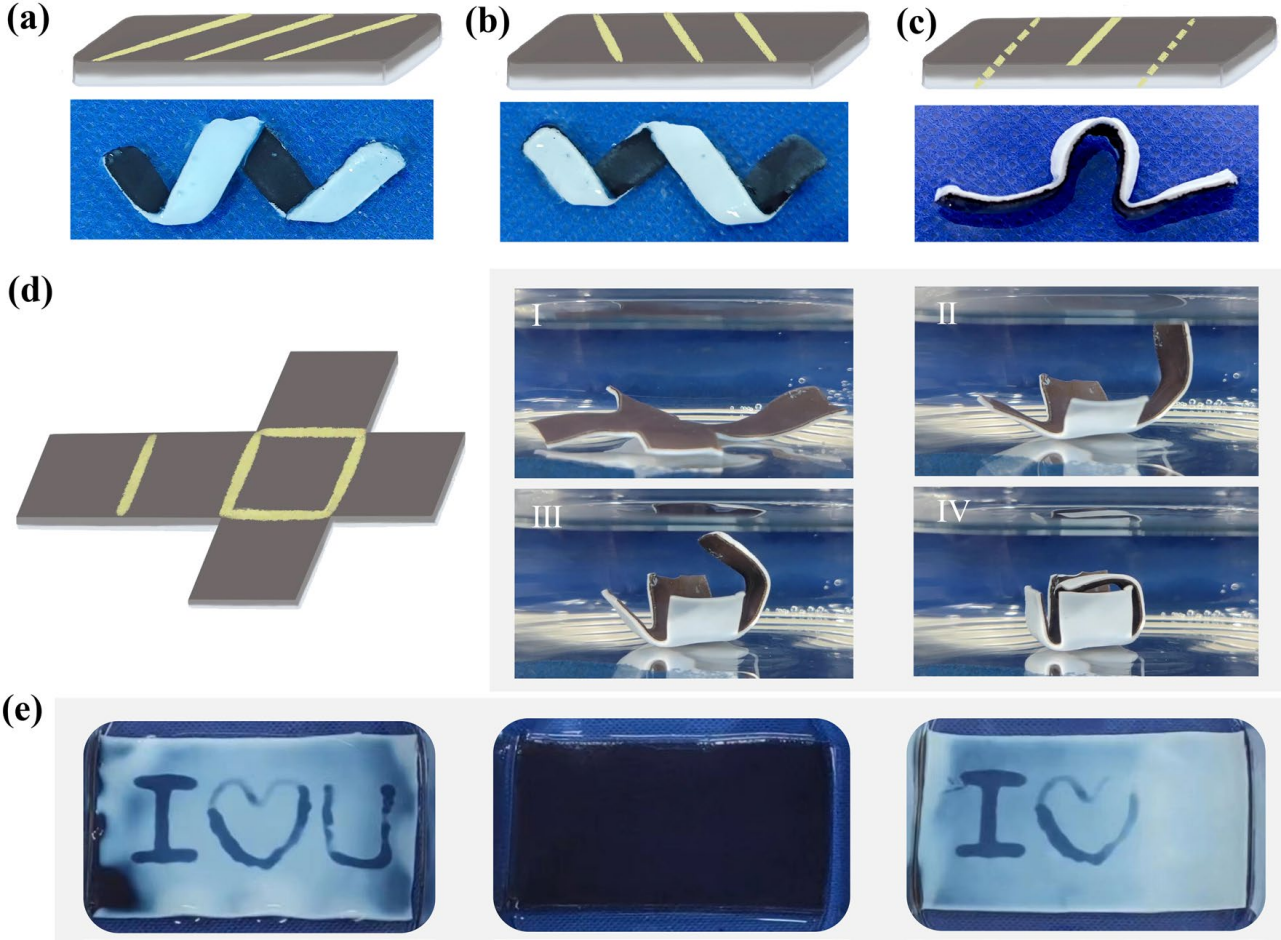
Programmable deformation and information display of PSM hydrogel. **a** Schematic illustration and photos of left oblique dip-coated with Ca^2+^. **b** Schematic illustration and photos of right oblique dip-coated with Ca^2+^. **c** Schematic illustration and photos of forward and reverse dip-coated with Ca^2+^. **d** Schematic illustration and photos of self-assembly folding cube. **e** Displaying and hiding information in PSM hydrogels

Shape-programmable PSM hydrogel actuators were further fabricated by patterning Ca^2+^ to locally crosslink SA, enabling various complex deformations under NIR light irradiation on demand. The deformation angle of the PSM hydrogel actuators can be easily controlled by adjusting the coating angle of Ca^2+^ (Fig. 5a, b). Interestingly, we observed that coating on the top and bottom surfaces resulted in different actuation directions. When Ca^2+^ was coated on the bottom surface, the PSM hydrogel bent toward the bottom, while coating Ca^2+^ on the top surface caused the hydrogel to bend toward the top (Fig. S12a, b). Therefore, by simultaneously patterning Ca^2+^ on the top and bottom surfaces, we obtained a more intricate deformation such as the character ‘Ω’ (Fig. 5c). Moreover, the PSM hydrogel actuators were capable of complex 3D folding movements. By creating the hydrogel according to the unfolding diagram of a cube and coating Ca^2+^ at the folding line (Fig. 5d), the hydrogel automatically folded and formed a closed cube when immersed in water at 50 °C (Movie S4). In addition, hydrogels can be used to display and hide information. Encoded messages can be written by coating Ca^2+^ (Fig. S13). The PSM hydrogel changed from hydrophilic to hydrophobic under thermal stimulation, with appearance changing from transparent to opaque, displaying the message 'I Love u' (Fig. 5e). After lowering the temperature, the PSM hydrogel reverted to its hydrophilic state, returning to its original transparent appearance, effectively concealing the message. Furthermore, the coordination reaction between EDTA solution and Ca^2+^ enabled message erasure, causing the letter 'U' to be effectively erased.

### Sensing Performance of PSM Hydrogel

**Fig. 6:**
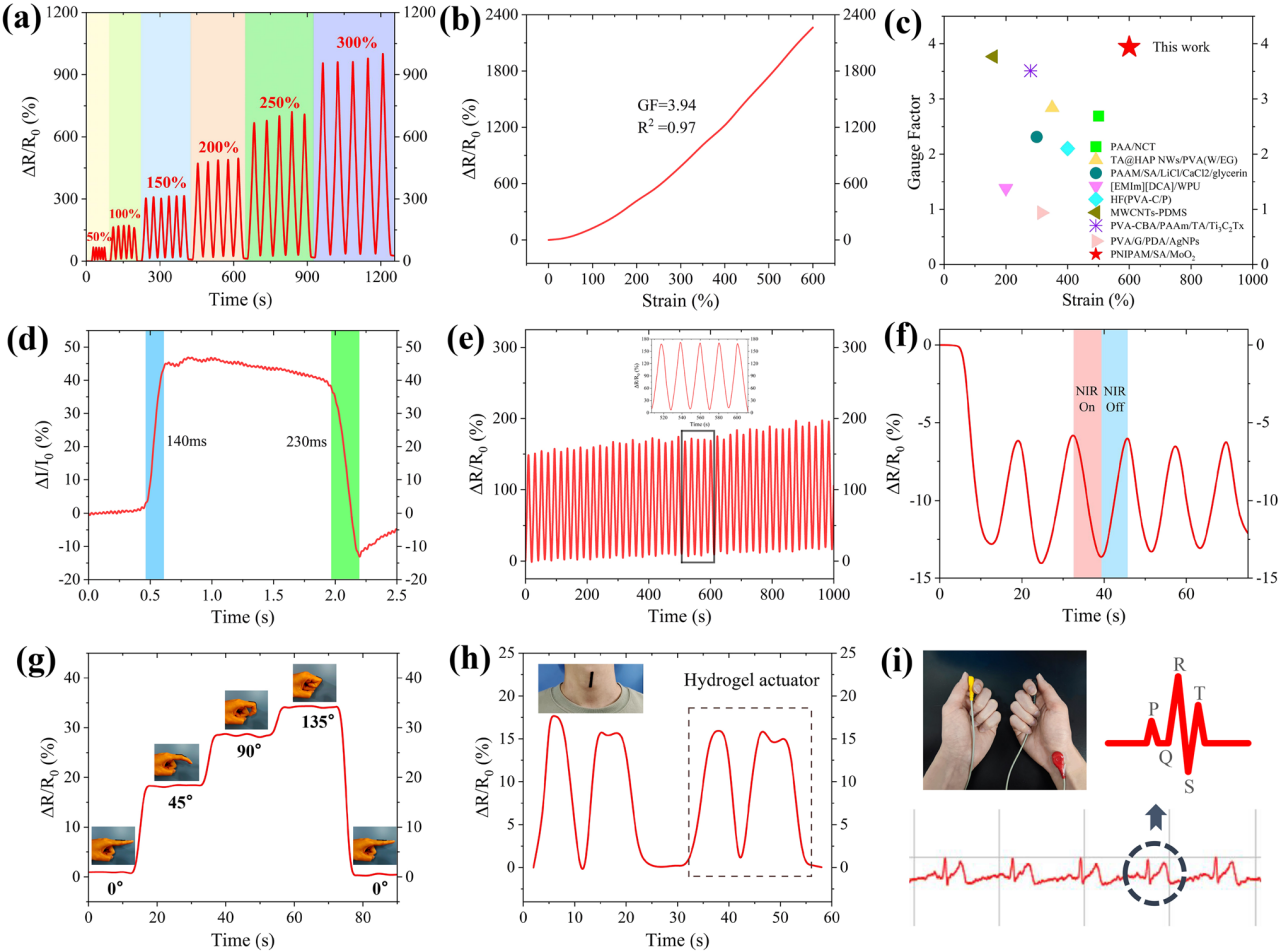
Sensing performance of PSM hydrogel. **a** RRCs of PSM hydrogel stretched with strains ranging from 50% to 300%. **b** RRCs of PSM hydrogel versus strain. **c** Comparison of Gauge factor and strain of hydrogels. Comparison of gauge factor within the strain of 600% of some typical reported strain sensors (see details in Table S2). **d** Response/recovery time of PSM hydrogels during stretch-relaxation. **e** RRCs of PSM hydrogels under 100% strain for 50 cycles. **f** Resistance changes for PSM hydrogel by periodically switching on and off of NIR radiation, with 5 cycles. **g** RRCs of PSM hydrogels from finger movement with different angles (0, 45°, 90°, 135°). **h** RRCs of PSM hydrogel adhered on the throat for the response of speaking "Hydrogel actuator". **i** The ECG signals detected by PSM hydrogel

In addition to ultrafast actuation, PSM hydrogels also exhibit impressive strain sensing and photothermal sensing capabilities. To further explore the sensing of the PSM hydrogel, we recorded the relative resistance change (RRC) of the hydrogel under different tensile strains. As shown in Fig. 6a, the RRC of PSM hydrogel gradually increased when the hydrogel was stretched from the original state to 50%, 100%, 150%, 200%, 250% and 300% with no hysteresis, showing an identifiable real-time response. To visualize the change in resistance, PSM hydrogel was used as a lead in the closed circuit to light the LED (Fig. S14). As the PSM hydrogel was stretched, the LED lamp gradually dimmed, and as the hydrogel returned to its original position and then folded, the LED brightened accordingly (Movie S5). Moreover, sensitivity is a crucial measure of sensing performance. Figure 6b shows that the gauging factor (GF) of PSM hydrogel, is defined as follows: (ΔR/R_0_)/ε, where ΔR is the RRC for different strains, R_0_ is the initial resistance and ε is the applied strain. Notably, the PSM hydrogel not only exhibited high sensitivity (GF = 3.94 within broad working strain range 600%), but also good stability with a high degree of linearity (R^2^ = 0.97), surpassing many previous reports (Fig. 6c and Table S2). Also, we assessed the responsiveness of PSM hydrogel by loading and unloading a small tensile strain, and demonstrate fast response times (140 ms) and recovery times (230 ms) (Fig. 6d). Furthermore, Fig. 6e shows the RRC of PSM hydrogel for 50 cycles with load-unload 100% strain, further demonstrating its good durability. In addition, PSM hydrogels demonstrated impressive photothermal responsive sensing properties. We based on the partial dehydration mechanism (Fig. S15a), where the aggregation of MoO_2_ nanoparticles under NIR irradiation reduces the hydrogel resistance (Fig. S15b). This allowed the resistance of PSM hydrogel can be changed rapidly and steadily by switching NIR light (Fig. 6f).

Thus, the unique sensing properties and high sensitivity of PSM hydrogels allow us to accurately detect various human signals. The PSM hydrogel was attached to joints for detecting human motion signals in real-time. As shown in Fig. 6g, the adhered hydrogel quickly detected the continuous change in finger bending as the angle of the finger changes from 0 to 135°. Once the finger rapidly changes to 0° again, the RRC also rapidly returns to its initial state, indicating highly sensitive response to motion detection. Also, other joint movements such as wrist, arm and leg can be detected based on the same principle (Fig. S16a–c). In addition, the PSM hydrogel can be used for human physiological signal detection by adhering PSM hydrogel to the throat. As shown in Fig. 6h, PSM hydrogel can detect the RRC generated by weak vibrations in the throat when the "Hydrogel actuator" was uttered. Meanwhile, PSM hydrogels can detect a continuous ECG signal by using a digital ECG acquisition module (Fig. 6i). The "PQRST" waveform for disease diagnosis was clearly visible on the ECG monitoring screen.

### Self-sensing and Bluetooth Interaction of PSM Hydrogel

**Fig. 7:**
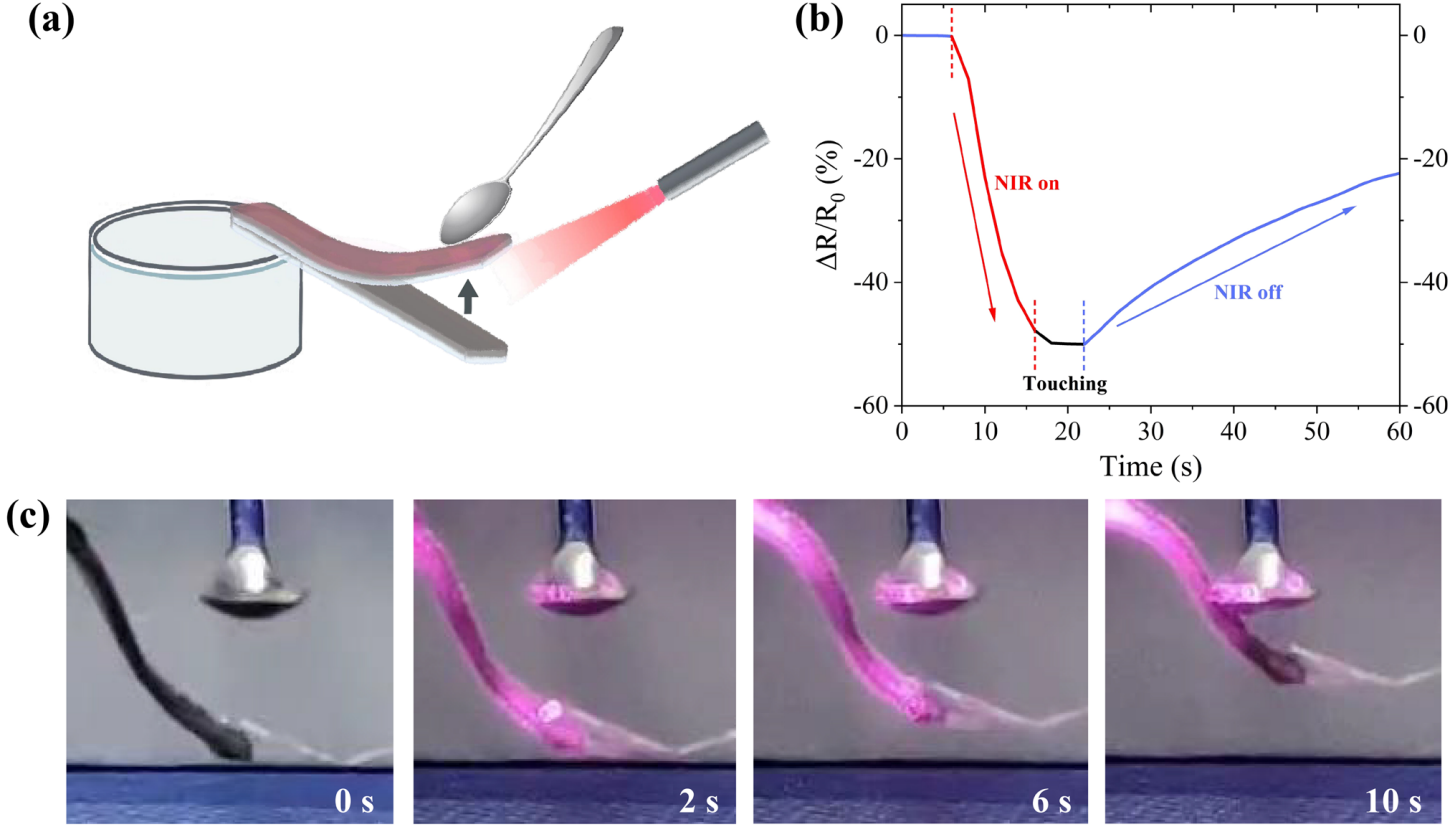
Self-sensing bioinspired artificial tongue. **a** Schematic illustration, **b** RRCs, and **c** the process of an artificial tongue touching a small spoon in response to NIR stimulation

Based on the exceptional combination of ultrafast actuation and high sensitivity of PSM hydrogels, we developed a self-sensing bioinspired artificial tongue, which sensed touching a spoon under NIR stimulation (Fig. 7a). As shown in Fig. 7b, c, the artificial tongue remained in a downward position without NIR light irradiation, resulting in no change in the resistance of the PSM hydrogel. When irradiated with NIR light, the artificial tongue started to bend upwards rapidly; while, the resistance of PSM hydrogel dropped sharply (Fig. 7b, red line). After 10 s, the artificial tongue gradually touched the spoon and the resistance of PSM hydrogel slowly decreased (Fig. 7b, black line). Finally, after turning off the NIR radiation, the artificial tongue softened back down and the resistance of PSM hydrogel gradually returned to its initial value (Fig. 7b, blue line). This successful demonstration exemplifies the achievement of self-sensing capabilities for bending and touching of PSM hydrogel.

**Fig. 8:**
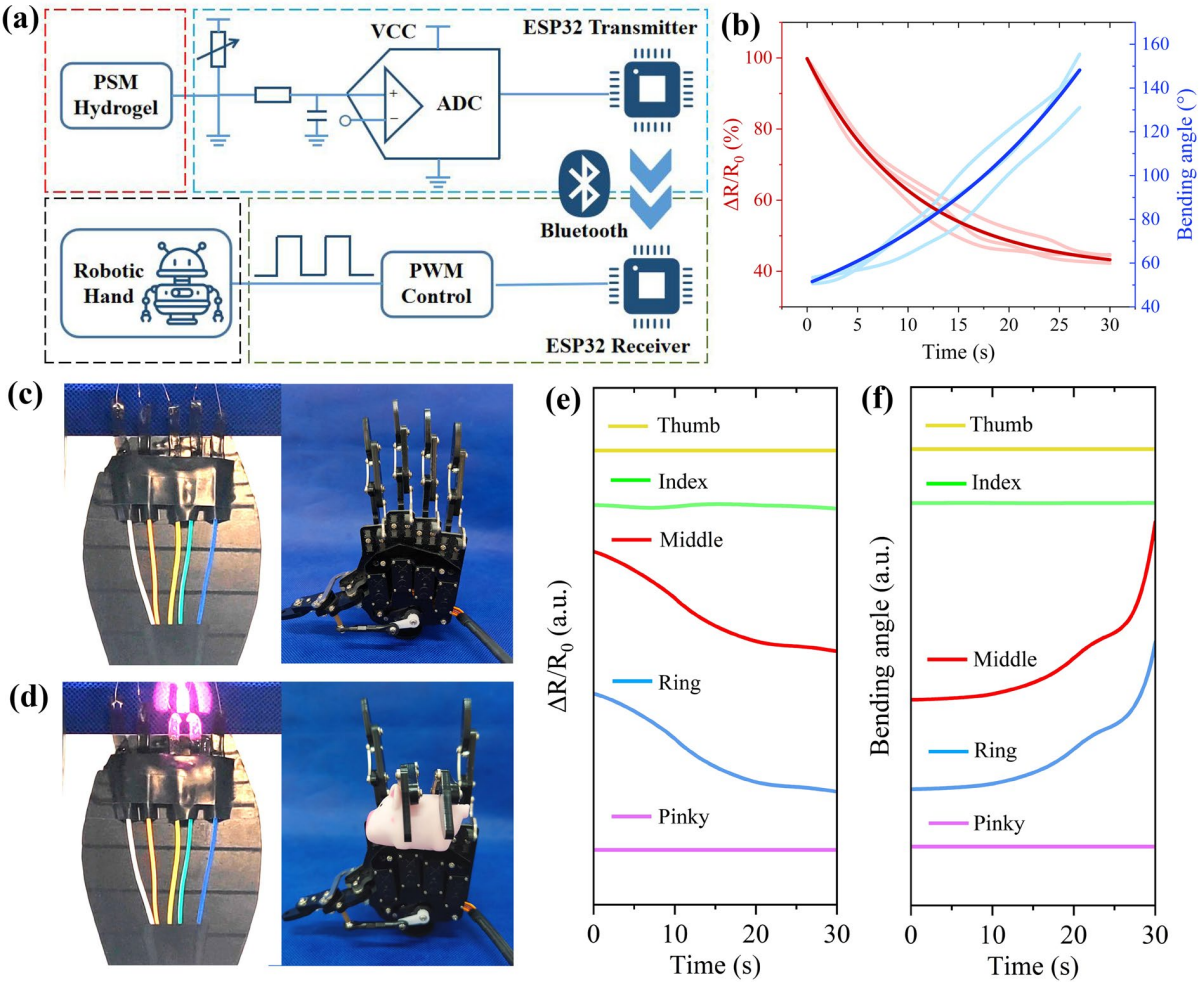
Soft-hard robot remote interaction system. **a** Schematic diagram. **b** Fitted curves of resistance and bending angle of PSM hydrogel are based on three test samples. **c** Before and **d** after NIR stimulation of self-sensing hydrogel actuators control a Bluetooth interactive robotic hand to grasp a pig doll. **e** Real-time resistance changes of five PSM hydrogel actuators under NIR stimulation. **f** Real-time bending angle changes of five robotic fingers in remote interaction

Furthermore, by leveraging IoT technology, we enabled remote interaction between soft and hard robots. Figure 8a outlined the system for soft PSM hydrogel actuators to control a hard robotic hand. The whole system comprises 4 main parts (Fig. S17a, b): (1) Self-sensing NIR-responsive stimulation of the PSM hydrogel. (2) Signal acquisition and transmission system, including signal acquisition module and Bluetooth transmission module. (3) Signal reception and control system, consisting of Bluetooth receiver module and actuation control module. (4) Controlled terminal for the robotic hand. Specifically, the self-sensing PSM hydrogel deformed and generated resistance changes after NIR stimulation. Then, the voltage signal changing in real-time from the PSM hydrogel was captured by the ESP32 chip transmitter and converted into a digital signal. Next, the signal was transmitted remotely via Bluetooth module to the ESP32 chip receiver and converted into the corresponding bending angle of the robotic hand. Finally, the servo motor was actuated by Pulse Width Modulation (PWM) and driven the robotic hand to bend, enabling remote interaction between the soft and hard robot. To further quantify and analysis PSM hydrogel self-sensing properties, the collected hydrogel resistance change data and bending angle data were nonlinearly fitted (Fig. 8b) as:

1 $$y_{R} = 59.3e^{{ - \frac{t}{10.3}}} + 40.1$$

2 $$y_{D} = 40.8e^{{\frac{t}{22.1}}} + 9.8$$

where *y*_*R*_, *y*_*D*_ are real-time resistance changes and bending angle changes of PSM hydrogel actuator, *t* is NIR irradiation time. Notably, we normalized the initial resistance of the five PSM hydrogels to reduce the error. Thus, *y*_*D*_ can be expressed as:

3 $$y_{D} = 40.8 \times \left( {\frac{59.3}{{y_{R} - 40.1}}} \right)^{0.47} + 9.8$$

As a result, we successfully established a quantitative relationship between the resistance and the bending angle of PSM hydrogel, which in turn actuated robotic hand to bend the same angle via Bluetooth interaction. Figure 8c, d shows PSM hydrogel precisely actuate the middle and ring fingers of robotic hand to bend and grasp the piggy doll (Movie S6). The resistance changes of the five PSM hydrogels and the corresponding angle changes of the five robotic fingers were clearly recorded in real-time (Fig. 8e, f). Thus, we have realized an IoT-based remote interactive system for NIR stimulation—self-sensing hydrogel actuator—Bluetooth interactive robotic hands. We believe that such soft materials with internal somatosensory actuation capabilities may open up new opportunities for remote control of intelligent robots.

## Conclusions

In conclusion, we have fabricated a bioinspired self-sensing actuated gradient hydrogel using a novel wettability-based strategy for remote interactions between soft and hard robots. The difference in wettability within the hydrogel arises from the deposition of MoO_2_ nanosheets during polymerization, leading to the formation of the gradient hydrogel. The presented hydrogel exhibited ultrafast actuation properties, showing ultrafast bending rate (21° s^−1^) in 50 °C water and good photothermal efficiency (3.7 ℃ s^−1^) under 808 nm NIR in 2 W. The hydrogel can not only be used as a gripper for fast grabbing (20 s), but also be made as soft robots including artificial iris and bionic jellyfish. Moreover, the hydrogel can be programmed to actuate and display information using Ca^2+^ patterned. In addition, the hydrogel has exceptional sensitivity including GF (3.94) in high stretchability (600%), fast response times (140 ms) and repeatable stability. The hydrogels were utilized as sensors for human motion and physiological signal detection. Combining actuation and sensing characteristics, a self-sensing bioinspired tongue was prepared. In particular, it was further combined with IoT to quantify the motion trajectory of the soft actuators, enabling a remote control system for NIR stimulus response—intelligent self-sensing soft robot—Bluetooth interactive robotic hands. In summary, this work proposes a novel and highly efficient method for the preparation of high performance multifunctional self-sensing actuated gradient hydrogels, paving the way for the development of next-generation intelligent interactive somatosensory soft material.

## Supplementary Information

Below is the link to the electronic supplementary material.

Supplementary file1 (DOCX 12717 kb)

Supplementary file2 (MP4 7566 kb)

Supplementary file3 (MP4 12625 kb)

Supplementary file4 (MP4 17429 kb)

Supplementary file5 (MP4 15632 kb)

Supplementary file6 (MP4 13718 kb)

Supplementary file7 (MP4 6773 kb)
