# Supplementary material for: Bioinspired Multifunctional Self-Sensing Actuated Gradient Hydrogel for Soft-Hard Robot Remote Interaction
Source: Nanomicro Lett. 2024 Jan 4;16:69. doi: 10.1007/s40820-023-01287-z (PMC10766940; doi:10.1007/s40820-023-01287-z)
Supplement: Supplementary file 1 — Supplementary file1 (DOCX 12717 kb) [file 40820_2023_1287_MOESM1_ESM.docx]

**Supplementary Information**

**Bioinspired multifunctional self-sensing actuated gradient hydrogel for soft-hard robot remote interaction**

He Liu^1^, Haoxiang Chu^1^, Hailiang Yuan^1^, Deliang Li^1^, Weisi Deng^1^, Zhiwei Fu^1^, Ruonan Liu^1^, Yiying Liu^1^, Yixuan Han^1^, Yanpeng Wang^1^, Yue Zhao^1^, Xiaoyu Cui^1, *^, Ye Tian^1, 2, *^

^1^College of Medicine and Biological Information Engineering, Northeastern University, Shenyang 110169, China

^2^Foshan Graduate School of Northeastern University, Foshan, 528300, China

Corresponding author:

Dr. Xiaoyu Cui, E-mail: cuixy@bmie.neu.edu.cn

Dr. Ye Tian, E-mail: tianye@bmie.neu.edu.cn


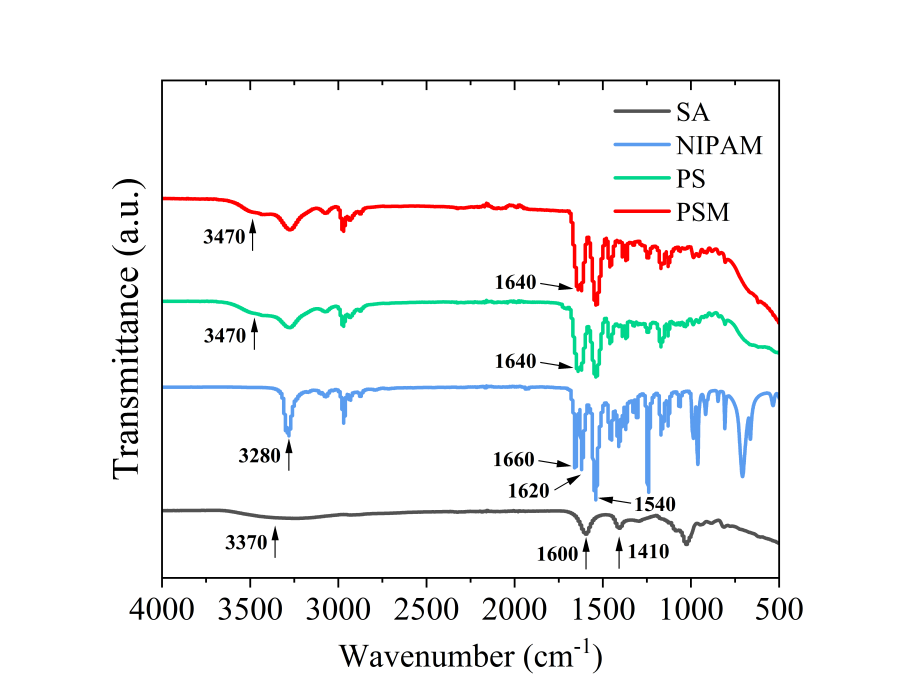


**Fig. S1** FTIR spectra of SA, NIPAM, PS hydrogel and PSM hydrogel


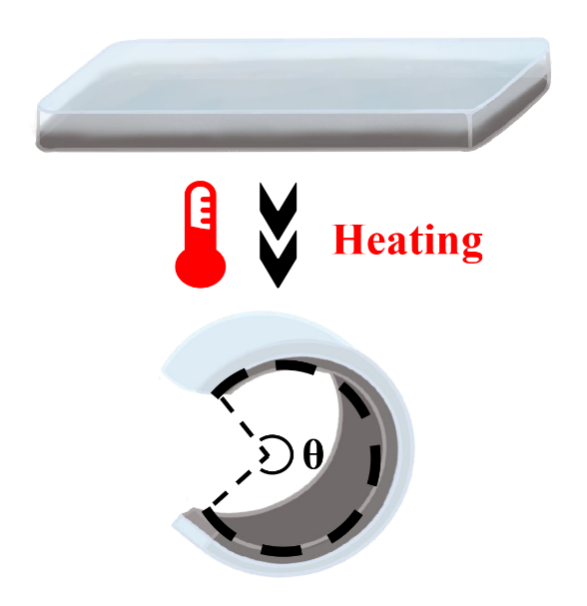


**Fig. S2** Scheme of measuring the bending angle (θ) of the hydrogel actuator


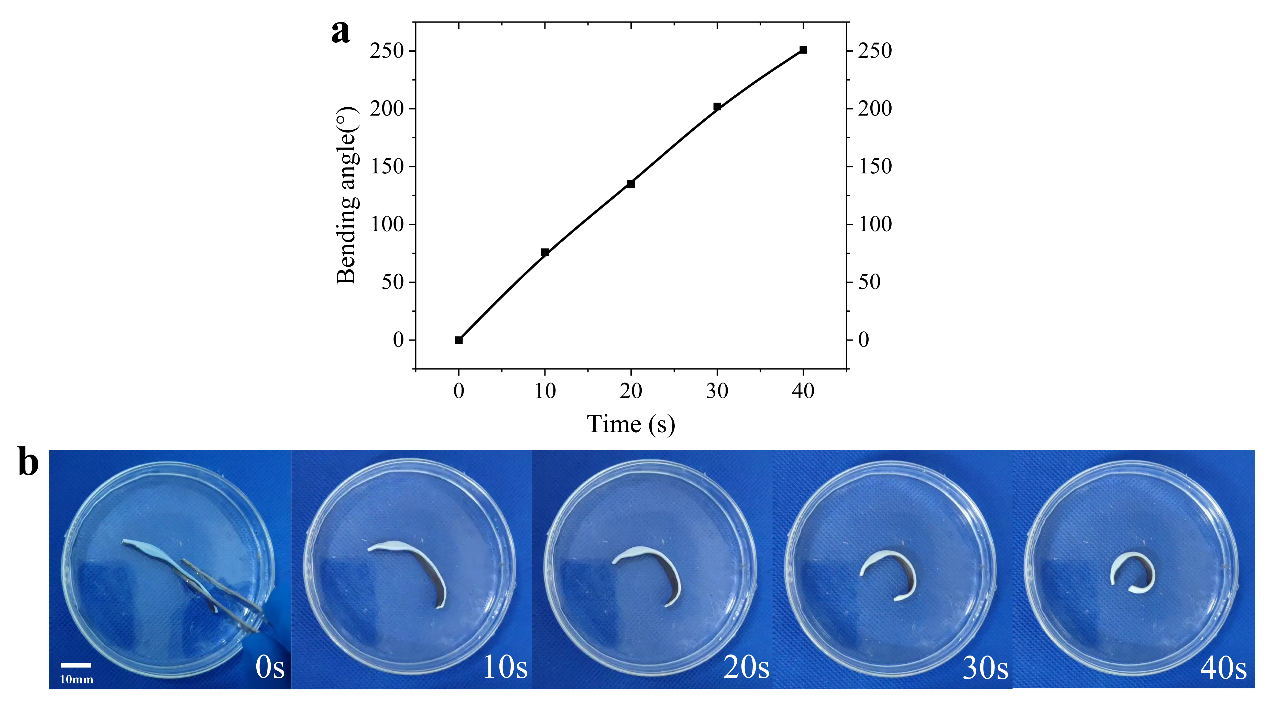


**Fig. S3** Thermo-responsive actuation **a** angle change curve, **b** physical diagram of the hydrogel without CaCl_2_ soaking in water at 50 °C


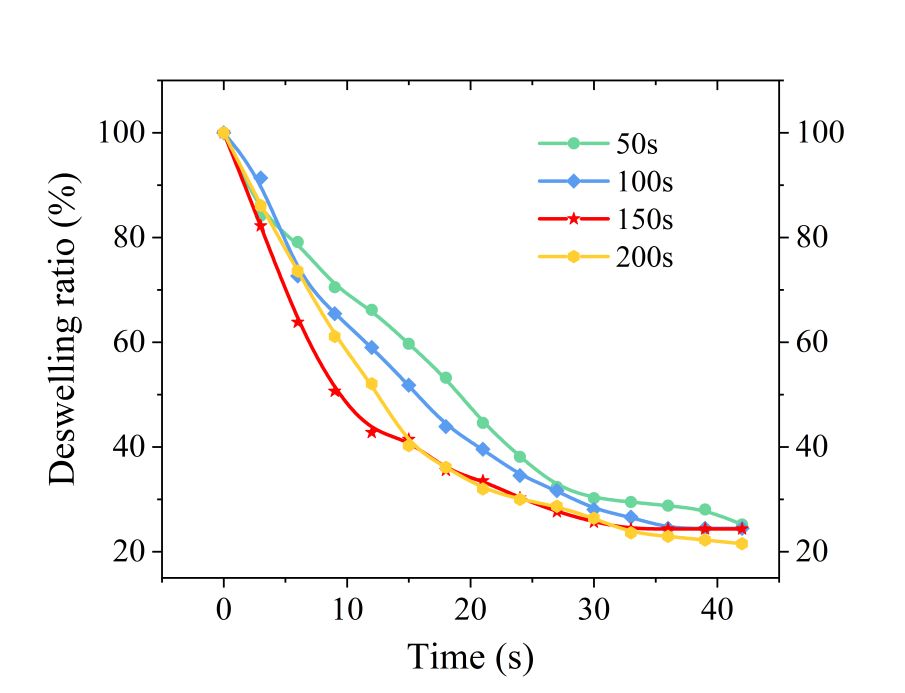


**Fig. S4** The deswelling ratio of PSM hydrogels soaked in CaCl_2_ for 50 s, 100 s, 150 s and 200 s


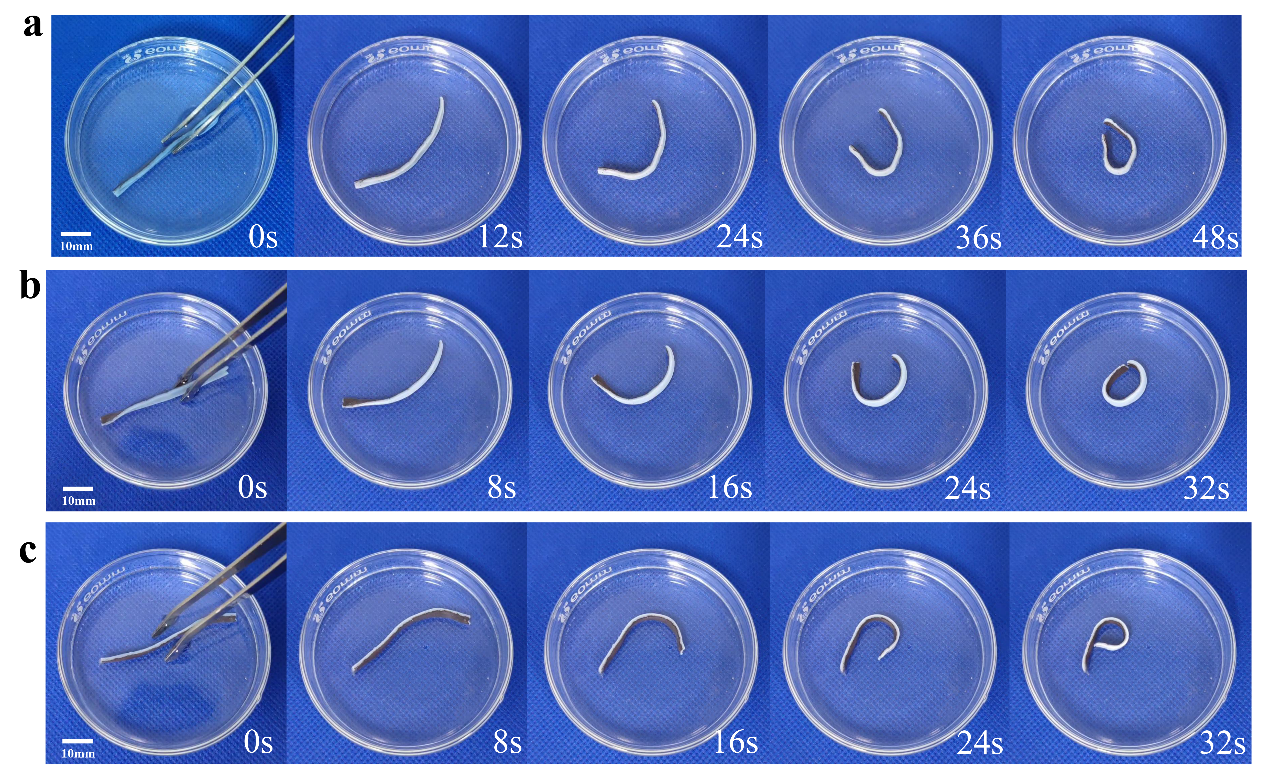


**Fig. S5** Thermo-responsive actuation of hydrogels after soaking CaCl_2_ for **a** 50 s, **b** 100 s, **c** 200 s in water at 50 °C


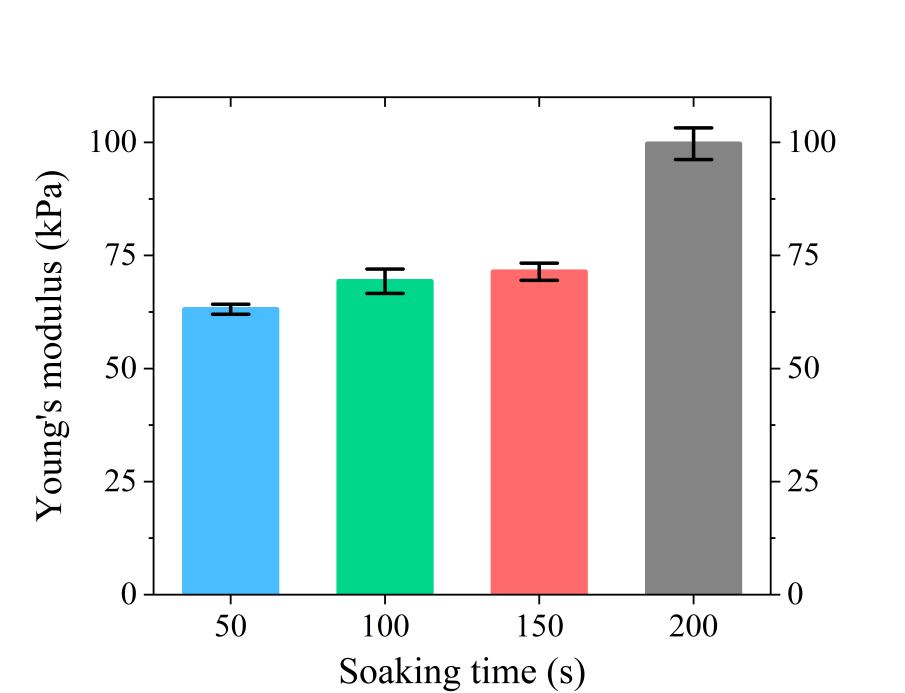


**Fig. S6** The Young's modulus of PSM hydrogels soaked in CaCl_2_ for 50 s, 100 s, 150 s and 200 s


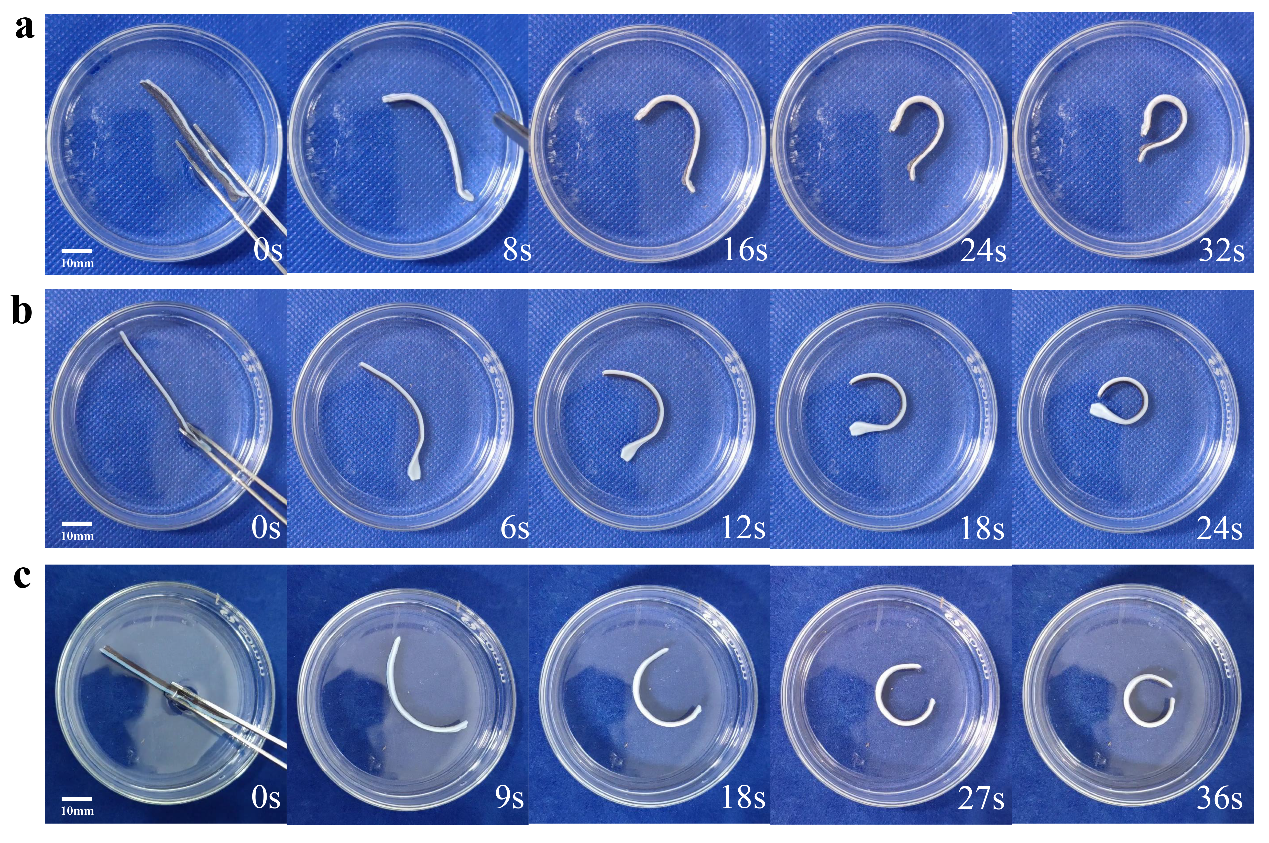


**Fig. S7** Thermo-responsive actuation of **a** PSM_0.2_, **b** PSM_0.4_, **c** PSM_0.8_ hydrogels in water at 50 °C


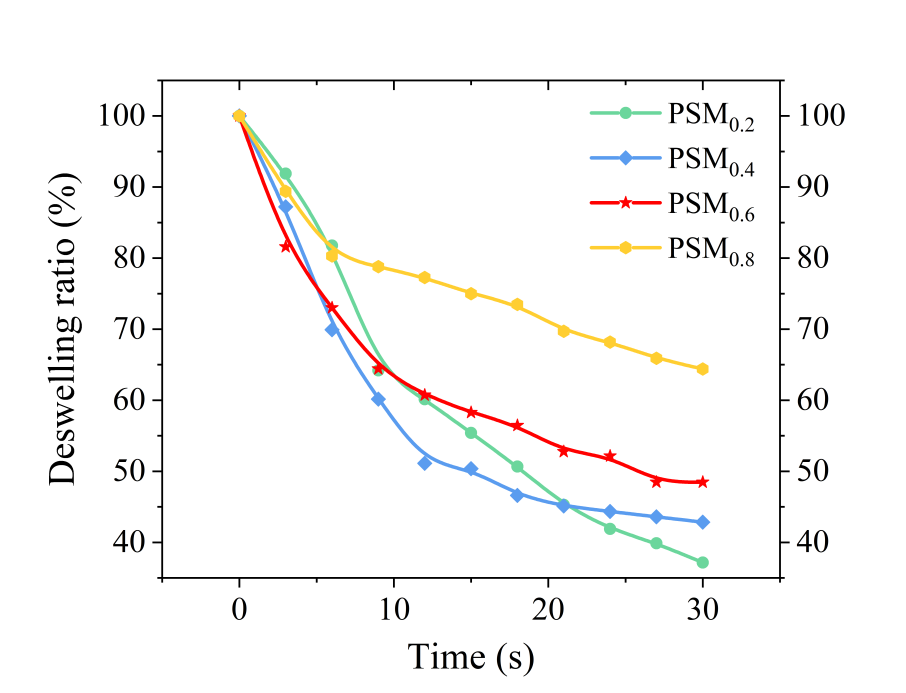


**Fig. S8** Deswelling curve of PSM hydrogel in water at 50 °C


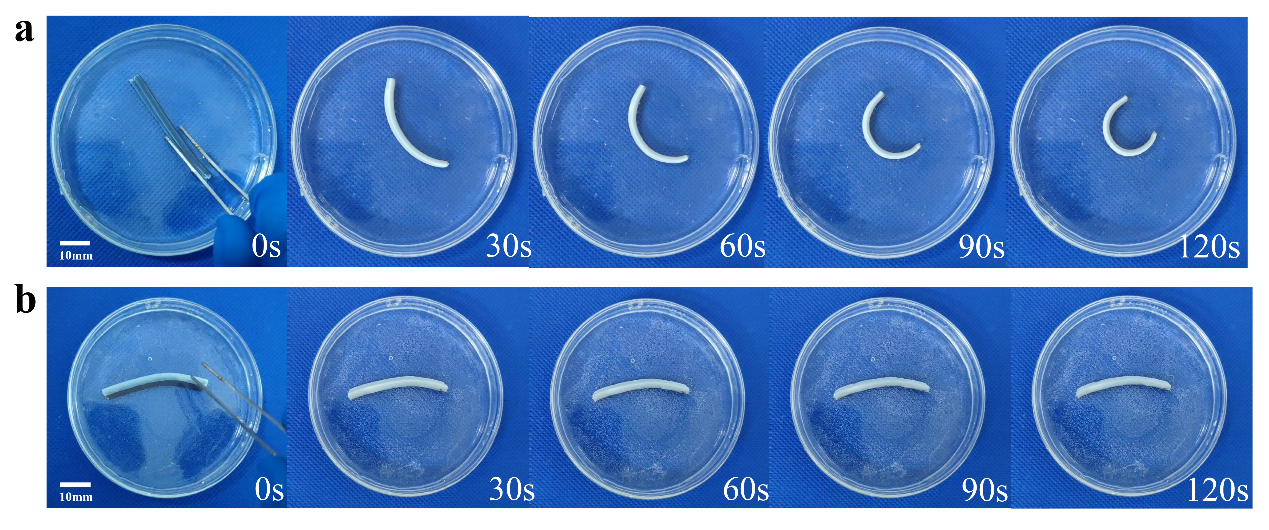


**Fig. S9** Thermo-responsive actuation of hydrogels with a thickness of **a** 2 mm and **b** 3 mm in water at 50 °C


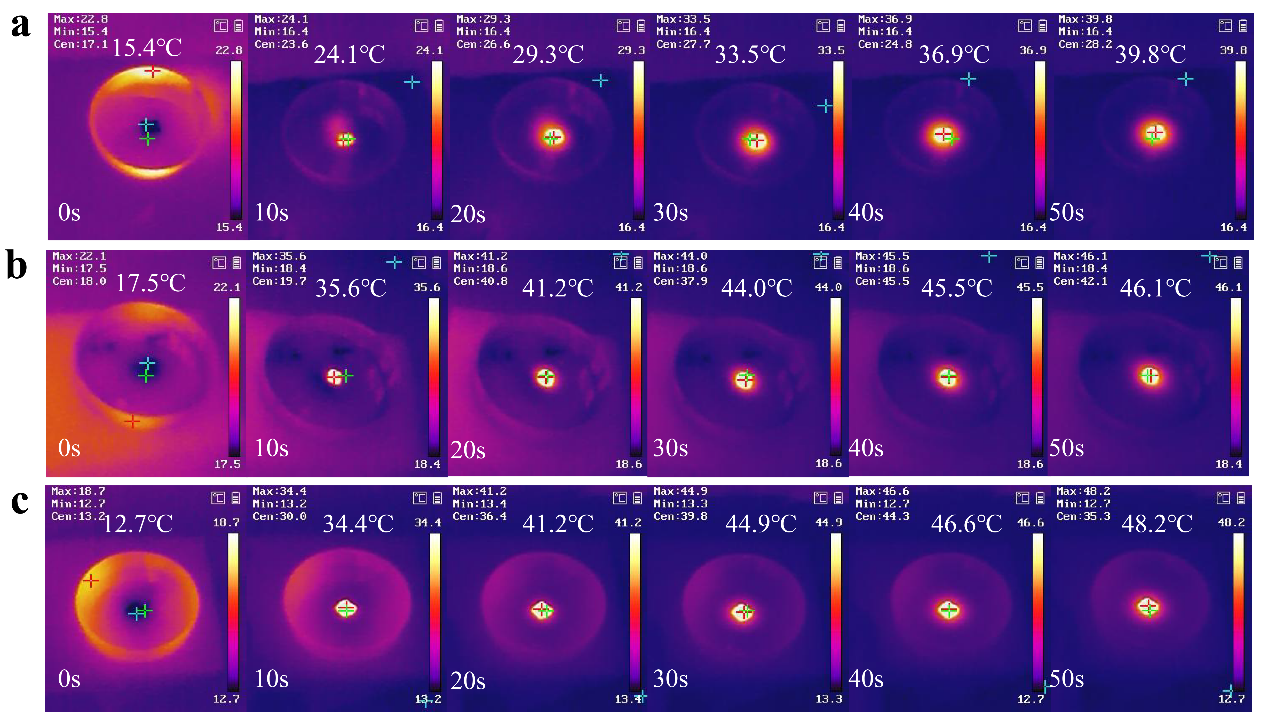


**Fig. S10** Temperature response changes of **a** PSM_0.2_, **b** PSM_0.4_ **c** PSM_0.8_ hydrogels under NIR light irradiation (808 nm, 1 W/cm^2^)


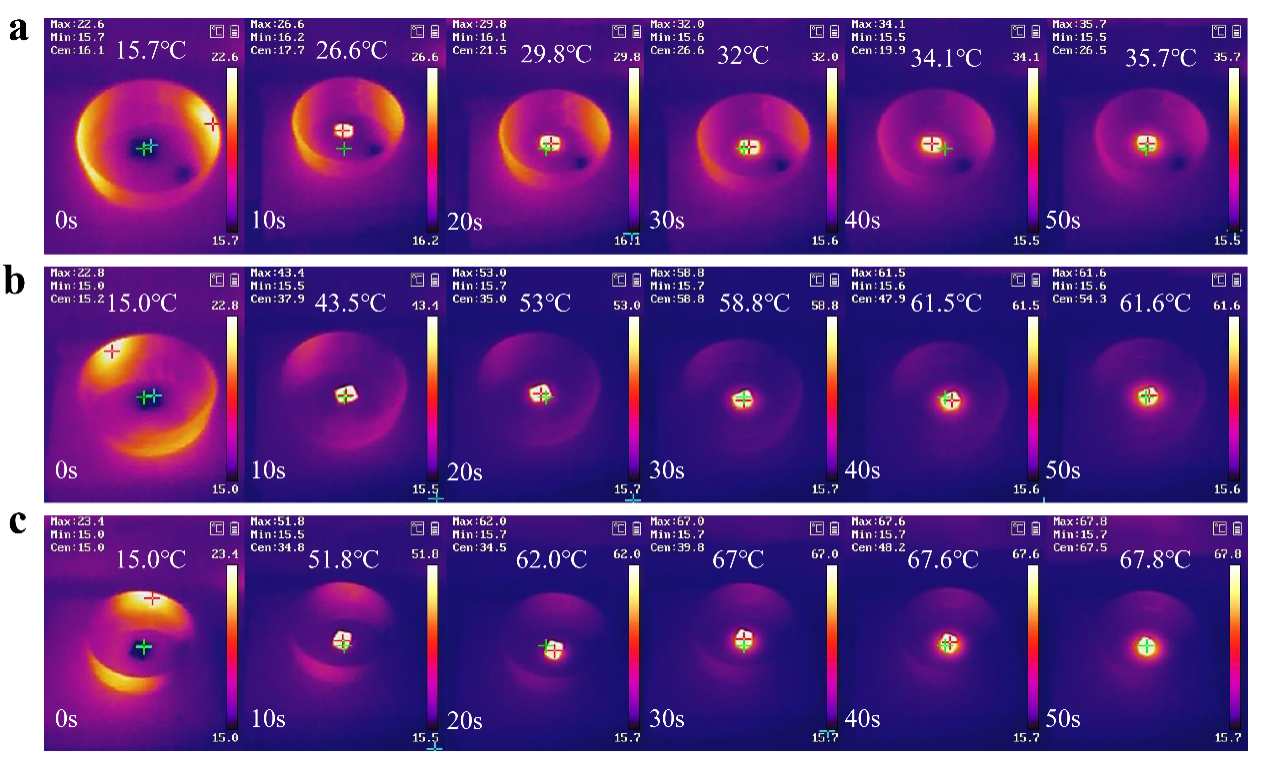


**Fig. S11** Temperature response changes of PSM_0.6_ hydrogel under 808 nm NIR light irradiation with **a** 0.5 W/cm^2^, **b** 1.5 W/cm^2^ , **c** 2 W/cm^2^


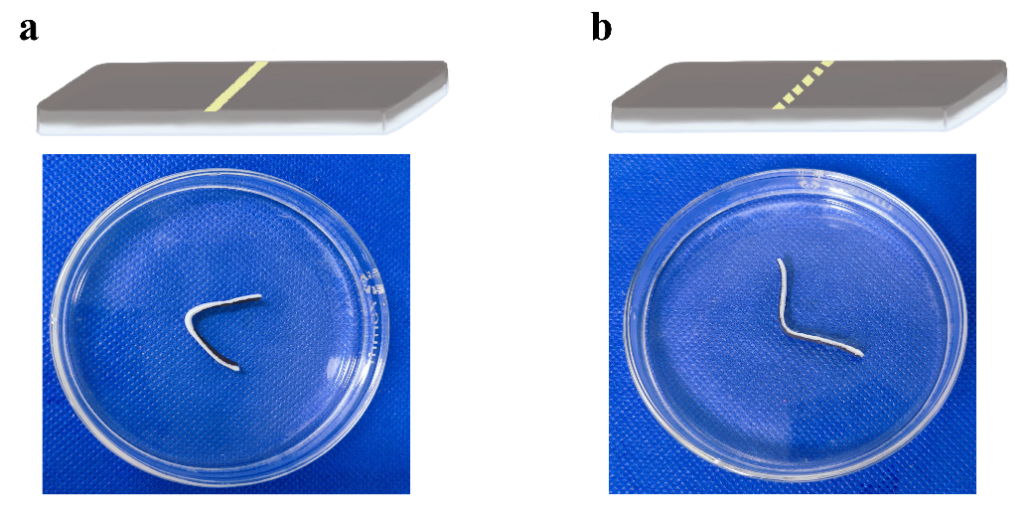


**Fig. S12** Shape deformation of PSM hydrogel actuators coating Ca^2+^ in the **a** bottom and **b** top directions


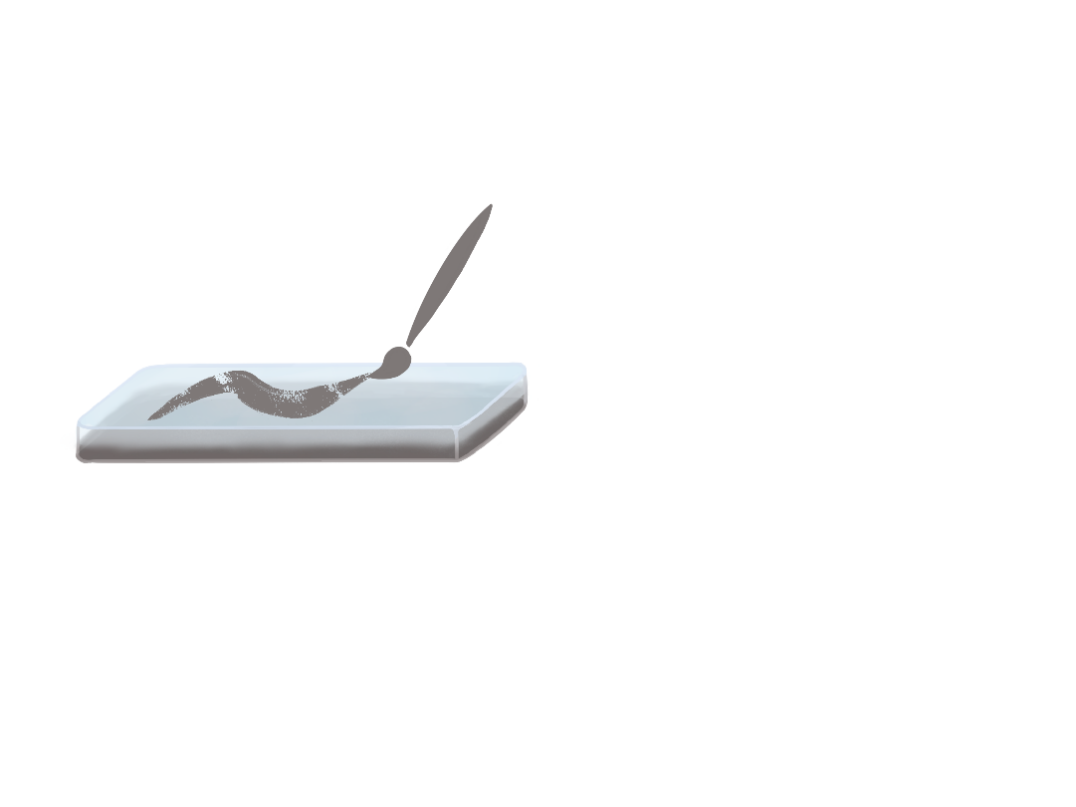


**Fig. S13** Diagram of encoded information written by coating Ca^2+^


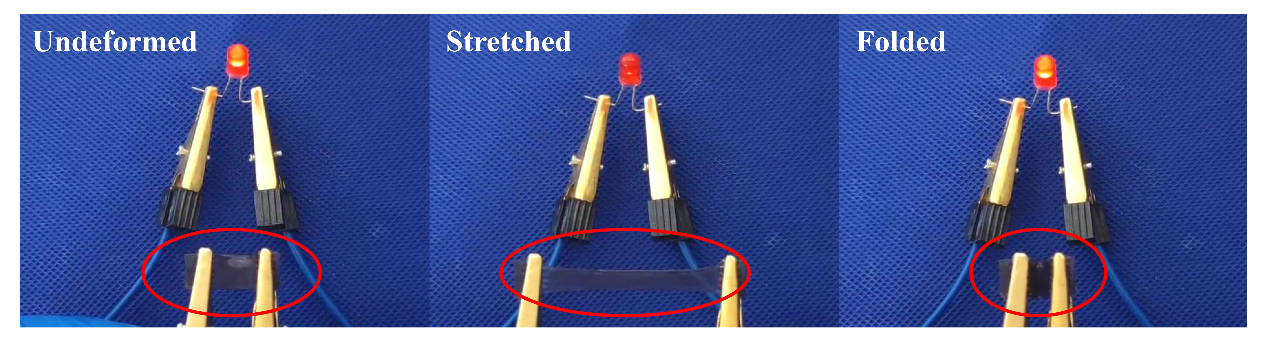


**Fig. S14** Brightness changes of LED in the undeformed, stretched and folded states of PSM hydrogels


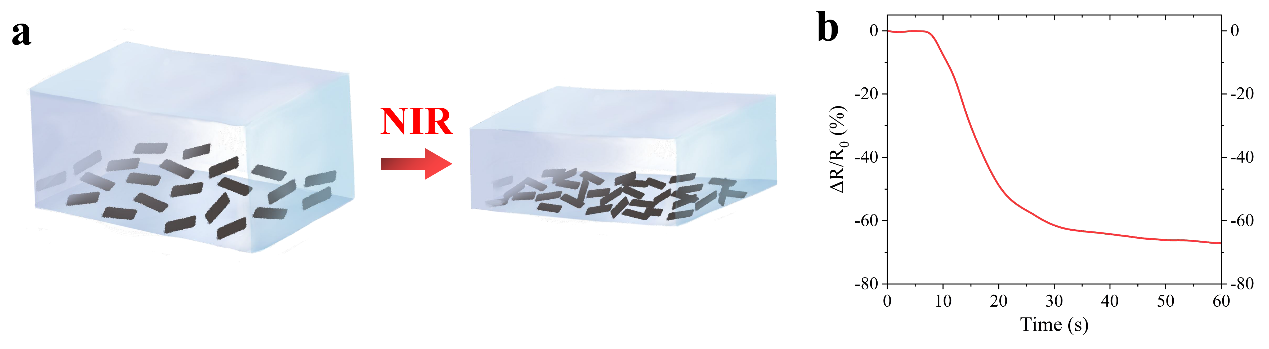


**Fig. S15 a** Photothermal sensing mechanism. **b** The relative resistance changes of PSM hydrogel under NIR irradiation


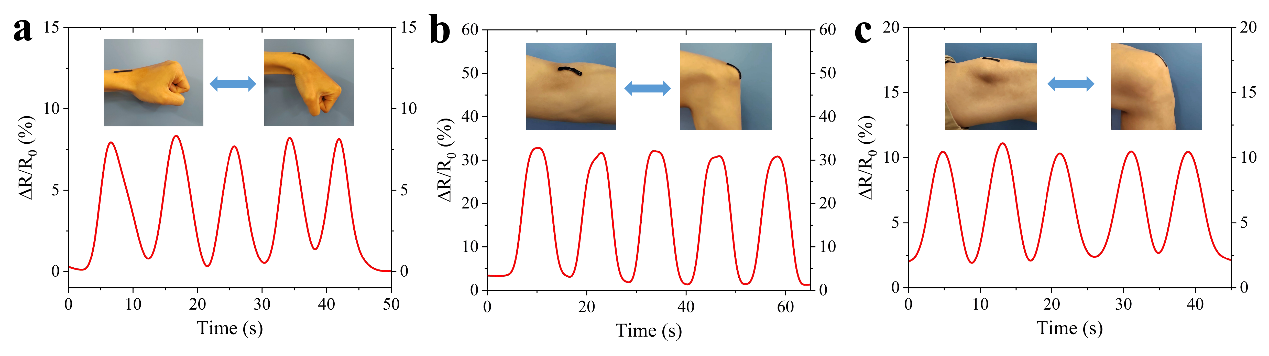


**Fig. S16** The relative resistance changes of PSM hydrogel under different joints include **a** wrist, **b** arm and **c** leg


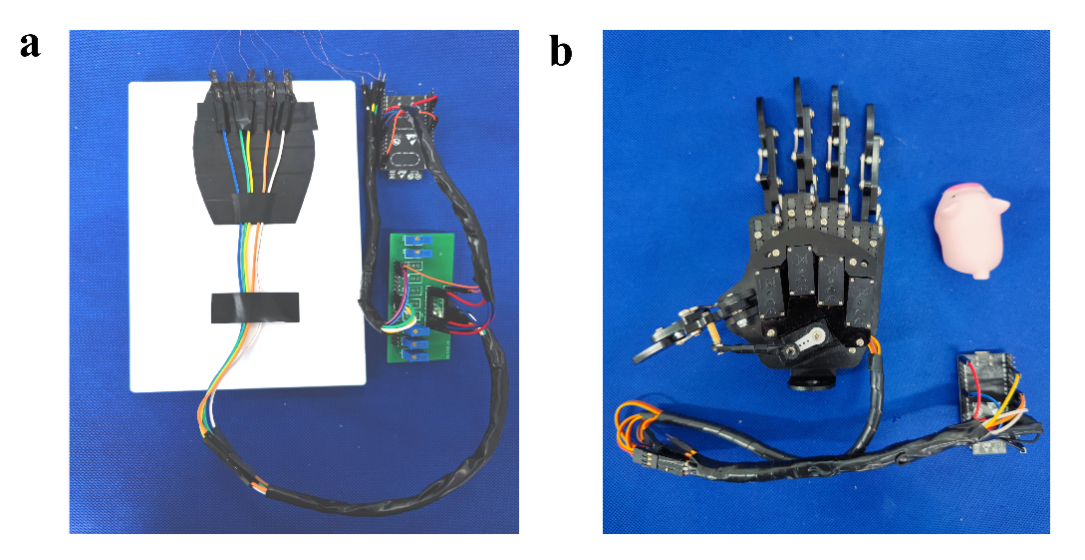


**Fig. S17** Hardware schematic of **a** PSM hydrogel and the signal transmission system, **b** Robotic hand and the signal receiver system

**Table S1.** Summarization for the bending speed and bending amplitude of hydrogel actuators

| **Material** | **Structure** | **Bending speed** | **Bending amplitude** | **Stimulation conditions** | **Sample size (mm^3^)** | **Ref** |
| --- | --- | --- | --- | --- | --- | --- |
| PNIPAM/  XLG/  HEA | Gradient | 9.8°/s | 206° | 50 ℃ water | 25 × 5 × 1 | [S1] |
| PNIPAM  /XLG | Gradient | 2.38°/s | 57.2° | 50 ℃ water | 25 × 5 × 1 | [S2] |
| P(NIPAM-co-SPA)/  TCNC | Gradient | 10.2°/s | 225° | 60 ℃ water | 25 × 5 × 0.3 | [S3] |
| PNIPAM/  XLG/NFC | Bilayer | 9°/s | 180° | 50 ℃ water | 65 × 7 × 2 | [S4] |
| PNIPAM/  PAAM-  PTCA | Bilayer | 6.1°/s | 305° | 45 ℃ water | —— | [S5] |
| PNIPAM/  XLG/  MoO_2_ | Bilayer | 3°/s | 90° | NIR 808 nm 0.8 W/cm^2^ | —— | [S6] |
| PNIPAM/PVA | Bilayer | 2.1°/s | 65° | NIR 808 nm 0.2 W/cm^2^ | —— | [S7] |
| PNIPAM/ SA/MoO_2_ | Gradient | 21°/s | 336° | 50 ℃ water | 50 × 10 × 1 | **This work** |

Notes: The comparison is only rough because these hydrogels have different sample sizes and stimulation conditions

**Table S2.** Comparison of the main conductive parameters of PSM hydrogel and conductive hydrogel strain sensors in previous mainstream work

| **Material** | **Strain (%)** | **Gauge Factor** | **Linear or Nonlinear** | **Conductive mechanism** | **Ref** |
| --- | --- | --- | --- | --- | --- |
| PAA/NCT | 500 | 2.69 | Linear | Ionic conduction | [S8] |
| TA@HAP NWs/PVA(W/EG) | 350 | 2.84 | Linear | Ionic conduction | [S9] |
| PAAM/SA/LiCl/CaCl_2_/glycerin | 0-100 | 0.45 | Nonlinear | Ionic conduction | [S10] |
|  | 200-300 | 2.31 |  |  |  |
| [EMIm][DCA]/WPU | 0-30 | 0.53 | Nonlinear | Ionic conduction | [S11] |
|  | 30-100 | 0.83 |  |  |  |
|  | 100-200 | 1.38 |  |  |  |
| HF(PVA-C/P) | 400 | 2.1 | Linear | Electronic conduction | [S12] |
| MWCNTs-PDMS | 160 | 3.77 | Linear | Electronic conduction | [S13] |
| PVA-CBA/PAAm/TA/  Ti_3_C_2_Tx | 0-80 | 1.2 | Nonlinear | Electronic conduction | [S14] |
|  | 80-150 | 1.57 |  |  |  |
|  | 150-220 | 2.34 |  |  |  |
|  | 220-280 | 3.51 |  |  |  |
| PVA/G/PDA/AgNPs | 0-70 | 0.94 | Nonlinear | Electronic conduction | [S15] |
|  | 70-315 | 0.13 |  |  |  |
| PNIPAM/SA/ MoO_2_ | 600 | 3.94 | Linear | Electronic conduction | **This work** |

**Supporting Movie 1** Thermo-responsive actuation behavior of hydrogels

**Supporting Movie 2** PSM hydrogel gripper grabs metal sheet from 50 °C water

**Supporting Movie 3** Bioinspired jellyfish swimming under NIR switch

**Supporting Movie 4** Self-assembly folding cube

**Supporting Movie 5** Brightness changes of LED in the undeformed, stretched and folded states of PSM hydrogel

**Supporting Movie 6** Remote interaction of soft-hard robot via IoT

**Reference**

[S1] Y. Tan, D. Wang, H. Xu, Y. Yang, X. L. Wang, F. Tian, P. Xu, W. An, X. Zhao, S. Xu. Rapid recovery hydrogel actuators in air with bionic large-ranged gradient structure. ACS Appl. Mater. Interfaces. **10**(46), 40125-40131 (2018). https://doi.org/10.1021/acsami.8b13235

[S2] Y. Tan, D. Wang, H. Xu, Y. Yang, W. An, L. Yu, Z. Xiao, S. Xu. A fast, reversible, and robust gradient nanocomposite hydrogel actuator with water-promoted thermal response. Macromol. Rapid Commun. **39**(8), e1700863 (2018). https://doi.org/10.1002/marc.201700863

[S3] J. Lin, Y. Han, Y. Cui, W. Zhao, C. Chang. Ionic coordination strengthening of temperature-driven gradient hydrogel actuators with rapid responsiveness. Compos. B. Eng. **245**, (2022). https://doi.org/10.1016/j.compositesb.2022.110210

[S4] Q. Zhao, Y. Liang, L. Ren, Z. Yu, Z. Zhang, F. Qiu, L. Ren. Design and fabrication of nanofibrillated cellulose-containing bilayer hydrogel actuators with temperature and near infrared laser responses. J Mater Chem B. **6**(8), 1260-1271 (2018). https://doi.org/10.1039/c7tb02853a

[S5] B. Y. Wu, X. X. Le, Y. K. Jian, W. Lu, Z. Y. Yang, Z. K. Zheng, P. Theato, J. W. Zhang, A. Zhang, T. Chen. Ph and thermo dual-responsive fluorescent hydrogel actuator. Macromol. Rapid Commun. **40**(4), e1800648 (2019). https://doi.org/10.1002/marc.201800648

[S6] Z. Sun, C. Wei, W. Liu, H. Liu, J. Liu, R. Hao, M. Huang, S. He. Two-dimensional moo(2) nanosheet composite hydrogels with high transmittance and excellent photothermal property for near-infrared responsive actuators and microvalves. ACS Appl. Mater. Interfaces. **13**(28), 33404-33416 (2021). https://doi.org/10.1021/acsami.1c04110

[S7] N. Chen, Y. Zhou, Y. Liu, Y. Mi, S. Zhao, W. Yang, S. Che, H. Liu, F. Chen, C. Xu, G. Ma, X. Peng, Y. Li. Conductive photo-thermal responsive bifunctional hydrogel system with self-actuating and self-monitoring abilities. Nano Res. **15**(8), 7703-7712 (2022). https://doi.org/10.1007/s12274-022-4394-3

[S8] X. Jing, P. Feng, Z. Chen, Z. Xie, H. Li, X.-F. Peng, H.-Y. Mi, Y. Liu. Highly stretchable, self-healable, freezing-tolerant, and transparent polyacrylic acid/nanochitin composite hydrogel for self-powered multifunctional sensors. ACS Sustainable Chem. Eng. **9**(28), 9209-9220 (2021). https://doi.org/10.1021/acssuschemeng.1c00949

[S9] J. Wen, J. Tang, H. Ning, N. Hu, Y. Zhu, Y. Gong, C. Xu, Q. Zhao, X. Jiang, X. Hu, L. Lei, D. Wu, T. Huang. Multifunctional ionic skin with sensing, uv‐filtering, water‐retaining, and anti‐freezing capabilities. Adv. Funct. Mater. **31**(21), 2011176 (2021). https://doi.org/10.1002/adfm.202011176

[S10] X.-y. Wang, H.-J. Kim. Ultra-stretchable dual-network ionic hydrogel strain sensor with moistening and anti-freezing ability. Prog. Org. Coat. **166**, 106784 (2022). https://doi.org/10.1016/j.porgcoat.2022.106784

[S11] B. Zhao, J. Yan, F. Long, W. Qiu, G. Meng, Z. Zeng, H. Huang, H. Wang, N. Lin, X. Y. Liu. Bioinspired conductive enhanced polyurethane ionic skin as reliable multifunctional sensors. Adv. Sci. **10**(19), e2300857 (2023). https://doi.org/10.1002/advs.202300857

[S12] Q. Zhang, Q. Wang, G. Wang, Z. Zhang, S. Xia, G. Gao. Ultrathin and highly tough hydrogel films for multifunctional strain sensors. ACS Appl. Mater. Interfaces. **13**(42), 50411-50421 (2021). https://doi.org/10.1021/acsami.1c15784

[S13] K. Zhang, C. Song, Z. Wang, C. Gao, Y. Wu, Y. Liu. A stretchable and self-healable organosilicon conductive nanocomposite for a reliable and sensitive strain sensor. J. Mater. Chem. C. **8**(48), 17277-17288 (2020). https://doi.org/10.1039/d0tc04719h

[S14] T. Gong, Z. N. Li, H. Liang, Y. Li, X. Tang, F. Chen, Q. Hu, H. Wang. High-sensitivity wearable sensor based on a mxene nanochannel self-adhesive hydrogel. ACS Appl. Mater. Interfaces. **15**(15), 19349-19361 (2023). https://doi.org/10.1021/acsami.3c01748

[S15] L. Fan, J. Xie, Y. Zheng, D. Wei, D. Yao, J. Zhang, T. Zhang. Antibacterial, self-adhesive, recyclable, and tough conductive composite hydrogels for ultrasensitive strain sensing. ACS Appl. Mater. Interfaces. **12**(19), 22225-22236 (2020). https://doi.org/10.1021/acsami.0c06091
